# Defining and Evaluating Microbial Contributions to Metabolite Variation in Microbiome-Metabolome Association Studies

**DOI:** 10.1128/mSystems.00579-19

**Published:** 2019-12-17

**Authors:** Cecilia Noecker, Hsuan-Chao Chiu, Colin P. McNally, Elhanan Borenstein

**Affiliations:** a Department of Genome Sciences, University of Washington, Seattle, Washington, USA; b Department of Computer Science and Engineering, University of Washington, Seattle, Washington, USA; c Blavatnik School of Computer Science, Tel Aviv University, Tel Aviv, Israel; d Sackler Faculty of Medicine, Tel Aviv University, Tel Aviv, Israel; e Santa Fe Institute, Santa Fe, New Mexico, USA; Mayo Clinic

**Keywords:** correlation, evaluation, metabolic modeling, metabolomics, microbiome

## Abstract

Correlation-based analysis of paired microbiome-metabolome data sets is becoming a widespread research approach, aiming to comprehensively identify microbial drivers of metabolic variation. To date, however, the limitations of this approach and other microbiome-metabolome analysis methods have not been comprehensively evaluated. To address this challenge, we have introduced a mathematical framework to quantify the contribution of each taxon to metabolite variation based on uptake and secretion fluxes. We additionally used a multispecies metabolic model to simulate simplified gut communities, generating idealized microbiome-metabolome data sets. We then compared observed taxon-metabolite correlations in these data sets to calculated ground truth taxonomic contribution values. We found that in simulations of both a representative simple 10-species community and complex human gut microbiota, correlation-based analysis poorly identified key contributors, with an extremely low predictive value despite the idealized setting. We further demonstrate that the predictive value of correlation analysis is strongly influenced by both metabolite and taxon properties, as well as by exogenous environmental variation. We finally discuss the practical implications of our findings for interpreting microbiome-metabolome studies.

**IMPORTANCE** Identifying the key microbial taxa responsible for metabolic differences between microbiomes is an important step toward understanding and manipulating microbiome metabolism. To achieve this goal, researchers commonly conduct microbiome-metabolome association studies, comprehensively measuring both the composition of species and the concentration of metabolites across a set of microbial community samples and then testing for correlations between microbes and metabolites. Here, we evaluated the utility of this general approach by first developing a rigorous mathematical definition of the contribution of each microbial taxon to metabolite variation and then examining these contributions in simulated data sets of microbial community metabolism. We found that standard correlation-based analysis of our simulated microbiome-metabolome data sets can identify true contributions with very low predictive value and that its performance depends strongly on specific properties of both metabolites and microbes, as well as on those of the surrounding environment. Combined, our findings can guide future interpretation and validation of microbiome-metabolome studies.

## Abstract (precis)

Identifying the key microbial taxa responsible for metabolic differences between microbiomes is an important step toward understanding and manipulating microbiome metabolism. To achieve this goal, researchers commonly conduct microbiome-metabolome association studies, comprehensively measuring both the composition of species and the concentration of metabolites across a set of microbial community samples and then testing for correlations between microbes and metabolites. Here, we evaluated the utility of this general approach by first developing a rigorous mathematical definition of the contribution of each microbial taxon to metabolite variation and then examining these contributions in simulated data sets of microbial community metabolism. We found that standard correlation-based analysis of our simulated microbiome-metabolome data sets can identify true contributions with very low predictive value and that its performance depends strongly on specific properties of both metabolites and microbes, as well as on those of the surrounding environment. Combined, our findings can guide future interpretation and validation of microbiome-metabolome studies.

## INTRODUCTION

Microbial communities have a tremendous impact on their surroundings, ranging from the degradation of environmental toxins (1) to the production of climate change-relevant metabolites (2). Host-associated communities, in particular, have a substantial impact on their hosts and often produce a diverse set of metabolites that interact with numerous host pathways. In humans, such microbiome-derived metabolites have been identified as factors contributing to a wide array of diseases, including heart disease (3), autism (4), nonalcoholic fatty liver disease (5), colon cancer (6), inflammatory bowel disease (7), and susceptibility to infection (8). Characterizing the ways microbial communities modulate their environments and the relationship between community structure and metabolic impact is therefore a major, timely, and complex challenge with promising implications for human health, as well as for environmental stewardship, agriculture, and industry.

In facing this challenge, perhaps the most important task is identifying specific community members that drive variation in metabolites of interest. Taxa responsible for observed metabolic differences across communities may be ideal targets for interventions aiming to modify metabolic phenotypes. Their identification, however, can be a daunting task. Complex microbial communities are often composed of hundreds or thousands of poorly characterized species, each with a unique and frequently unknown complement of metabolic capacities. Even when multiple species are known to possess the potential to synthesize or degrade a metabolite of interest, the metabolic activity of each species (and, consequently, its contribution to metabolic variation) may be different (9). Moreover, community ecology, interspecies interactions, and nutrient availability (e.g., via diet) can all regulate and influence the metabolic activity of each species, rendering the link between community members and metabolic products extremely complex and challenging to infer (10–12).

To address this challenge and to identify community members that play an important role in metabolic variation, a growing number of studies are now comprehensively assaying multiple facets of community structure across samples, including, most notably, taxonomic and metabolite compositions (13). For example, many recent studies have combined fecal microbiome profiling with metabolomics and dietary data to characterize metabolic interactions between diet and the human gut microbiome (11, 14–16). Others have applied these technologies to investigate the links between taxonomic shifts and metabolic phenotypes in nongut body sites, including the vaginal and oral microbiomes (17, 18), as well as non-human-associated microbial communities (19, 20). These are just a few examples of a plethora of recent microbiome-metabolome studies, investigating the metabolic effects of microbiome variation in the contexts of chronic and infectious disease, antibiotic resistance, agriculture, precision medicine, nutrition, fermented food science, and more (21–30). Such multi-omic studies are also a major focus of several large-scale initiatives to study both host-associated and environmental microbiomes (31, 32).

Given the taxonomic and metabolomic profiles obtained via such microbiome-metabolome assays, the vast majority of studies rely on simple univariate correlation-based analyses to link variation in community ecology to variation in metabolic activity (11, 17, 21, 33–37). Such analyses specifically aim to identify species whose abundance across samples is correlated with the concentration of metabolites, often assuming that highly significant correlations reflect a direct mechanistic link between the taxon and the metabolite in question. It is not uncommon for these studies to further suggest that positive correlations imply synthesis and negative correlations imply degradation or that targeting the microbe in question could modulate the concentrations of the metabolites with which it is correlated. In one recent example, a large microbiome-metabolome study of inflammatory bowel disease patients and controls posited that an association between a microbial and a metabolite that is observed among both patient and control subgroups is evidence of a mechanistic relationship corresponding to direct metabolism, selection, or ecological inhibition of other species (36). Similarly, another study characterizing the microbiome and metabolome in Spleen-yang-deficiency syndrome (37) concluded that a positive correlation between *Bacteroides* and mannose likely resulted from extracellular degradation of mannan into mannose by that taxon; also, a study of antibiotic perturbations to the microbiome and metabolome stated that the presence of several weak positive and negative correlations between genera and arginine supported the conclusion that arginine levels may be affected by many community members with high functional redundancy (33).

Yet, to date, the extent to which a correlation-based analysis effectively detects direct metabolic relationships between taxa and metabolites has been unclear. Obviously, a strong correlation between the abundance of a certain species and the concentration of a metabolite across samples might reflect direct synthesis or degradation of the metabolite by that species but might also arise due to environmental effects, precursor availability, selection, random chance, or co-occurrence between species. Similarly, cross-feeding, external host processes, and differing enzymatic regulation characteristics can mask a correlation even when the species does in fact contribute to observed metabolite variation. Indeed, previous studies have suggested that microbe-metabolite correlations must have a high rate of false positives (38), and recent experimental studies pairing microbiome-metabolome correlation analysis with *in vitro* monoculture validations found anecdotally that several observed correlations were in fact false positives or that the hypothesized mechanistic relationship could not be confirmed (36, 39). The limitations of correlation analysis have also been discussed and well characterized in other data types (see, for example, references 40 and 41). Importantly, however, the extent of such limitations in the context of microbiome-metabolome studies, the ways they are shaped by microbial community metabolism, and their impact on data interpretation in this context have not been systematically evaluated. Such context-specific validation has been recently highlighted as an important growth area in genomics and bioinformatics (42).

Two crucial challenges hinder a comprehensive and systematic evaluation of correlation-based analysis. The first challenge is the lack of a rigorous general definition of a microbe’s contribution to metabolite variability. While establishment of the main taxonomic contributors to metabolite variation may be straightforward for specialized, well-characterized metabolites that are synthesized by just a single taxon, it can be much less clear for metabolites that can be synthesized (and/or degraded or modified) by many different taxa in the community. Ideally, we would hope to identify which taxa have the largest effects on the levels of a metabolite, while accounting for their covariance in abundance and activity. The second challenge is the absence of ground truth data on the nature of microbe-metabolite relationships. While limited data on the taxa driving metabolite shifts can be obtained from comparative mono- and coculture studies (39, 43, 44), large-scale and comprehensive data sets that link species and metabolite abundances in the context of a complex community, for which the precise impact of each species on observed metabolite variation is known, are currently not available.

In this report, we address these two challenges, combining a novel framework for quantifying microbial contributions with model-based simulated data sets. Specifically, we first introduce a generalizable and rigorous mathematical framework for decomposing observed metabolite variation and quantifying the contribution of each community member to this variation based on uptake and secretion fluxes. Second, we use a dynamic multispecies genome-scale metabolic model to simulate the metabolism of microbial communities of various complexity and to generate idealized data sets of paired taxonomic and metabolomic abundances, with complete information on metabolite fluxes, microbial growth, interspecies interactions, and environmental influences. Applying our mathematical framework to these simulated data sets, we then compare calculated contribution values to observed taxon-metabolite correlations and evaluate the ability of correlation-based analyses to identify key microbial contributors. We additionally investigate factors that shape the relationship between community composition and metabolism in depth and analyze the data to identify specific properties and mechanisms that can impact the performance of microbiome-metabolome correlation studies.

Notably, given the objectives of this study, we intentionally focus on characterizing microbiome-metabolome relationships in a model-based, tractable, and well-defined setting. Indeed, our metabolic model may not perfectly capture all of the complex and diverse mechanisms that are at play in host-associated communities; however, considering the scope of this study, accurately recapitulating the metabolism of a specific community may not be crucial. Rather, for our analysis, we want our simulated data to capture broad trends observed in naturally occurring microbial ecosystems, as indeed has been demonstrated for several similar dynamic simulation frameworks (45–48). Moreover, utilizing this model-based approach allows us to dissect the relationship between community composition and metabolic phenotypes without the complexities inherent in *in vivo* communities (including spatial heterogeneity, measurement error, intermicrobial signaling, or strain-level variation). To this end, we first analyze simulated data sets from a set of “toy”-model, simplified microbiomes and then compare our findings with those from a more complex and realistic human gut-based data set. Analyzing the ability of a correlation-based analysis to detect true microbial drivers of metabolite variation in simplified, best-case settings provides a baseline for the expected performances of such analyses in real microbiome-metabolome studies.

## RESULTS

### Quantifying the impact of individual microbial species on variation in metabolite concentrations.

In this study, we consider a microbial community as an idealized system, consisting of a population of multiple microbial species in a shared, well-mixed, biochemical environment. Each species takes up necessary metabolites from the shared environment, performs a variety of metabolic processes to promote its growth, and secretes certain metabolites back into the shared environment. We additionally assume that certain nutrients flow into the environment and that microbial cells and metabolites are diluted over time. These processes can represent, for example, the inflow of dietary nutrients and the transit through the gut in the context of the gut microbiome. For simplicity, we primarily consider constant inflow and dilution rates, as in a chemostat setting. Accordingly, a microbiome-metabolome study can be conceived as analyzing a set of several such communities (at a certain point in time), all with differing compositions of microbial species and correspondingly differing environmental metabolite concentrations. We focus initially on a naive and highly controlled setting with identical nutrient inflow across all microbiomes but later examine the impacts of differences in nutrient inflow between communities.

Given this setting, we first sought to establish a rigorous and quantitative framework for defining the impact of each microbial species (or any taxonomic grouping) in the community on the variation observed in the concentration of a given metabolite across community samples. We focused on species that directly modulate the environmental concentration of a given metabolite via synthesis or degradation, ignoring indirect effects via, for example, the synthesis of a precursor substrate that could impact the metabolic activity of other species. We noted that the total concentration of any metabolite in the environment can be represented as the sum of cumulative synthesis or degradation fluxes of this metabolite mediated by each of the *n* species in the community, as well as cumulative environmental fluxes (e.g., total nutrient inflow and dilution). Formally, the concentration of a given metabolite *M* can therefore be expressed as a sum of *n* dependent random variables *m_i_*_,_ where each *m_i_* value denotes the overall synthesis or degradation of the metabolite by each species (with *m_i_* values of >0 for synthesis and *m_i_* values of <0 for degradation), along with an additional random variable *m*_env_, denoting the overall impact of environmental processes, as follows:

$$M=\overset{n}{\underset{i=1}{\sum }}m_{i}+m_{\text{env}}$$

As discussed above, in analyses of microbiome-metabolome data sets, the goal is often to identify taxa responsible for changes in the concentration of a metabolite of interest across a set of samples. Accordingly, here we quantify the contribution of each species to the variance in the concentration of that metabolite across samples. Specifically, in the formulation above, var(*M*) depends on the variance of the constituent microbial and environmental factors, as well as on the covariance between these components. This variance can then be linearly separated into *n + 1* terms, representing the contribution of each species (denoted *c_i_*), and of any environmental nutrient fluxes (denoted *c*_env_), to the total variation in the metabolite as follows:

$$\text{var}\left(M\right)=\overset{n}{\underset{i=1}{\sum }}c_{i}+c_{\text{env}};c_{i}=\text{var}\left(m_{i}\right)+\underset{j\neq i}{\sum }\text{cov}\left(m_{i},m_{j}\right)+\text{cov}(m_{i},m_{\text{env}})$$

Each contribution value is also equivalent to the covariance between the fluxes of the corresponding factor with the total concentration (see Materials and Methods). If the nutrient inflow is constant across samples, its effect can be ignored and its contribution to the variance *c*_env_ is 0. Additionally, while the concentration of metabolites is also affected by dilution, in a chemostat setting, its effect can be accounted for in the calculation of each contribution, as it depends strictly on the fixed dilution rate and on previous metabolite concentrations (see Materials and Methods). Finally, in order to compare species contributions across metabolites and to represent the relative share of the total variance of a given metabolite that is attributable to species *I*, we defined the relative contribution to variance c^i of each species *i* to metabolite *M* by normalizing contribution values by the metabolite’s total variance as follows:

c^i=civar(M) 

This framework for calculating microbial contribution values provides a systematic measure of the causal impact of each taxon on observed variation in the environmental concentration of each metabolite, distilling the effect of complex ecological and metabolic interactions to a concise and interpretable set of quantities. Moreover, the obtained contribution profile represents a linear decomposition of observed metabolic variation, wherein the sum of contributions of all species equals the observed variance in the metabolite. A large positive contribution value therefore indicates that the species in question was responsible for a substantial share of the observed variation in the concentration of the metabolite. Notably, under the definition provided above, contribution values can be negative when the activity of a given species has large negative covariances with the activities of other community members. Such negative contribution values indicate that the secretion or uptake of that metabolite by the species mitigates the impact of the activity of others. Correspondingly, contribution values can be greater than 1, reflecting scenarios in which a species in fact generates more variation of this metabolite than is ultimately observed but the impact is mitigated by other species.

It is also worth noting that our analytical decomposition of contributions to variance is mathematically equivalent to calculating the Shapley values for the variance in metabolite concentrations (see Materials and Methods; see also Fig. S1 in the supplemental material). Shapley value analysis is a game theory technique that defines an individual’s contribution to a collective outcome and has been shown to be the only general definition that is efficient, linear, and symmetric and that assigns zero values to null contributors (49). A similar, Shapley value-based approach was recently applied to address the related problem of identifying the primary taxonomic contributors to differential functional abundances in metagenomic data (50).

10.1128/mSystems.00579-19.2 FIG S1 Shapley values are equivalent to analytically calculated variance contributions. (A) Plot of contribution values calculated analytically versus those obtained from a Shapley value-based permutation analysis using 15,000 species orderings (see Materials and Methods), for all 52 analyzed metabolites in our main simulated 10-species dataset. Axes are on a log_10_ scale. (B) Plot of the total sum of squared error between Shapley values calculated using permuted species subsets and our analytically calculated variance contributions, for all metabolites in the 10-species dataset. With increasing numbers of permutations, and therefore increasingly precise contribution estimates, the difference between these values approaches 0. Download FIG S1, TIF file, 0.2 MB. Copyright © 2019 Noecker et al. 2019 Noecker et al. This content is distributed under the terms of the Creative Commons Attribution 4.0 International license.

### A multispecies metabolic model for generating complex microbiome-metabolome data.

We next set out to generate a large-scale data set of microbiome-metabolome profiles with complete information about metabolite uptake and secretion fluxes. To this end, we used a multispecies metabolic model to simulate the growth, dynamics, metabolism, and environment of a simple microbial community. As noted above, in this model (and in the resulting data set), we aim to recapitulate broad metabolic trends and the complex relationships that can occur between microbial taxa and metabolites rather than to perfectly capture the metabolism and behavior of a specific microbial ecosystem. This model is based on a previously introduced genome-scale framework for modeling the metabolism of multispecies communities and for tracking the metabolic activity of each community member over time (51, 52; see also references [Bibr B53][Bibr B54][Bibr B55]). Briefly, this framework assumes that each species optimizes its growth selfishly given available nutrients in the shared environment and predicts the metabolic activity for each species in small time increments using flux balance analysis (FBA) (56). After each increment, the model uses the predicted metabolic activities of the various species to update the biomass of each species and the concentration of metabolites in the shared environment (and hence potentially impacting the growth and metabolism of other species in subsequent time steps). Importantly, this model enables the natural emergence of metabolic competition and exchange between species, as well as selection for taxa with the most efficient growth characteristics in a given nutrient environment. Full details of this model and simulation parameters can be found in Materials and Methods.

**FIG 1:**
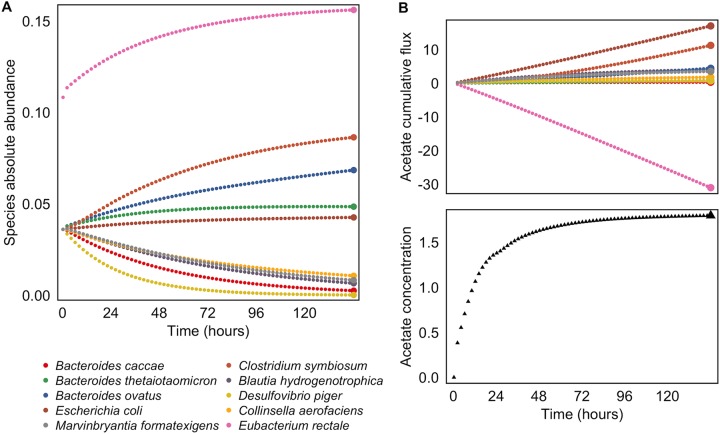
Simulating multi-omic data with a dynamic multispecies genome-scale framework. (A) Community species abundances throughout a single 10-species simulation run. Abundances were quantified in units of microbial biomass. In this simulation, community composition was initialized with a high relative abundance of Eubacterium rectale. For visual clarity, only every eighth time step is illustrated. Species abundances at the final time point (highlighted with larger colored circles) were used for calculating species-metabolite correlations. (B) Cumulative secretion and uptake of acetate by each community member, throughout the same simulation run as that illustrated in panel A. Acetate was synthesized by several species and consumed by E. rectale over the course of the simulation. Cumulative fluxes at the final time point (highlighted with larger colored circles) were used for calculating species contributions to metabolite variation. The bottom plot illustrates the resulting environmental concentration of acetate at each time point. The metabolite concentration at the final time point (highlighted with a larger black triangle) was used for calculating species-metabolite correlations.

We first specifically modeled a simplified gut community that had previously been explored experimentally (57). This community includes 10 representative gut species, spanning the major clades found in the human gut and collectively encoding the key metabolic processes taking place in this environment, including breakdown of complex dietary polysaccharides, amino acid fermentation, and removal of fermentation end products via sulfate reduction and acetogenesis. Genome-scale metabolic models of these 10 species were obtained from the AGORA (assembly of gut organisms through reconstruction and analysis) collection (48)—a recently introduced set of high-quality gut-specific metabolic reconstructions. To mimic the experimental gnotobiotic mouse setting (57), we simulate growth in a chemostat, with a nutrient inflow mimicking the content of a standard corn-based mouse chow and a dilution rate consistent with mouse transit time and gut volume (see Materials and Methods). While maintaining this nutritional environment, we systematically explored the landscape of possible community compositions, adjusting the initial relative abundance of each species from 10% to 60% (with a consistent total abundance equal to the community carrying capacity), which facilitated clearly interpretable mechanistic links between initial species abundances and final metabolite concentrations, resulting in a total of 61 different community compositions. For most analyses, we simulated growth for 144 h (as 576 15-min time steps). For most community compositions considered, this simulation duration consisted of an initial stabilization period leading to near-steady-state conditions, with little change in community composition (Fig. 1A). Notably, across the various simulations, some species maintained high abundances throughout the course of the simulation whereas others reverted to lower levels.

**FIG 2:**
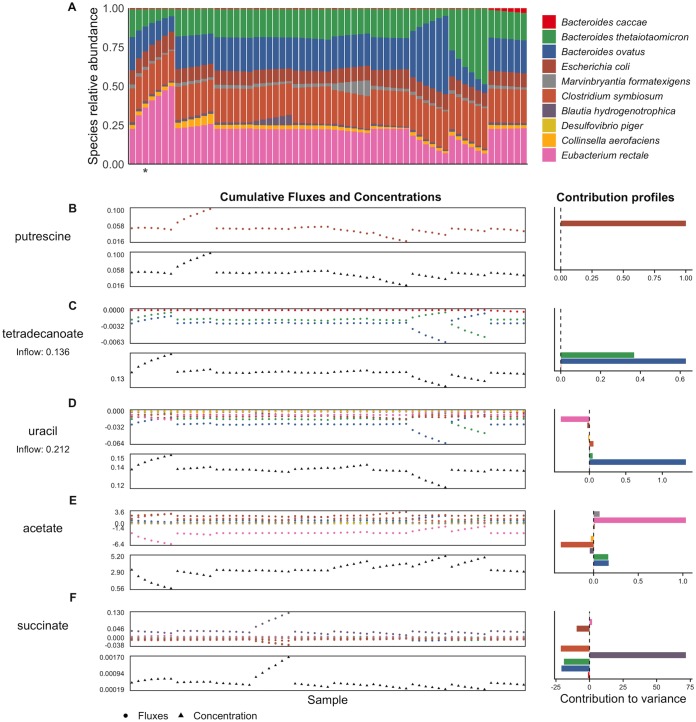
Species abundances, cumulative fluxes, and contributions to variance in metabolite concentrations in the 10-species simulated data set. (A) The data set of species abundances at the final time point of 61 simulation runs. Each bar represents a simulation run, with the colors indicating relative abundance of each species. The abundance profile from the simulation runs highlighted in Fig. 1 is indicated with an asterisk. (B to F) For five example metabolites, namely, putrescine (B), tetradecanoate (C), uracil (D), acetate (E), and succinate (F), the upper plot shows the total cumulative secretion or uptake of that metabolite by each species across all 61 simulation runs (or samples), and the lower plot shows the corresponding environmental concentration at the final time point. The bar plots on the right show the contribution values for each species and metabolite calculated from the flux values and describing the linear contribution of each species to the overall metabolite variance.

Throughout the course of each simulation, we recorded the abundances of each species, the rates of secretion and uptake of each metabolite by each species (as well as internal reaction fluxes), and the concentration of each metabolite in the environment (Fig. 1), thereby obtaining a comprehensive data set representing species composition, metabolic activities, and metabolite concentrations across 61 different communities. To mirror the typical structure of a microbiome-metabolome cross-sectional data set, we specifically considered the abundances of species and the concentrations of metabolites in the environment at the end of each simulation (i.e., after the final time point; see Fig. 1). Of the 68 metabolites present in the nutrient inflow, 60 exhibited at least some variation across communities, as did 18 additional microbially produced metabolites. Metabolite variation was generally low (median coefficient of variation, 0.021), reflecting the uniform nutrient environment, and yet 25 metabolites (32%) did have a coefficient of variation greater than 0.1. For downstream analysis, we excluded metabolites without substantial measurable variance across samples, filtering those with variance at or below the 25th percentile. This resulted in a data set of 52 variable metabolites, of which 14 were purely microbially produced metabolites, 9 were microbially produced but also present in the nutrient inflow, and 29 were introduced only through the nutrient inflow. Of these 52 variable metabolites, 47 were utilized by at least one member of the community (including 18 that were cross-fed in at least one simulation). The final species compositions and the final concentrations of several key metabolites across all simulations are shown in Fig. 2.

Exploring this data set, we found that species compositions and metabolite concentrations exhibited complex patterns and biologically plausible distributions (Fig. S2) (58), although the initial species abundances did result in an unusual variance structure (and see also our discussion of this structure below). Nevertheless, examining the obtained metabolic processes, we found that several processes known to occur in the mammalian gut were replicated by our simulations, including, for example, conversion of acetate to butyrate by Eubacterium rectale (59) and production of key microbial metabolites such as 4-aminobutyric acid (GABA), indole, and succinate. Cross-feeding relationships (corresponding to 18 metabolites) were also observed frequently, including cross-feeding of 6 amino acids whose exchange is widespread in host-associated microbiota (60). We additionally ran several sets of simulations with introduced fluctuations in the nutrient inflow concentrations (described in a separate section below) and found that the resulting species compositions partially recapitulated the diet responses observed by Faith et al. (57) (see Text S1 in the supplemental material).

10.1128/mSystems.00579-19.1 TEXT S1 Additional findings on (i) the recapitulation of experimental findings by simulation data; (ii) an alternative definition of contribution values based on steady-state fluxes; (iii) an alternative definition of key contributors based on contribution value magnitude; (iv) effects of simulation duration and *V*_max_ parameter on contribution and correlation results; (v) an analysis of features distinguishing correlated key contributor pairs from false-positive correlations; and (vi) additional effects of inflow fluctuations on contribution and correlation profiles. Download Text S1, PDF file, 0.2 MB. Copyright © 2019 Noecker et al. 2019 Noecker et al. This content is distributed under the terms of the Creative Commons Attribution 4.0 International license.

10.1128/mSystems.00579-19.3 FIG S2 Distributions of species and metabolite abundances. (A and B) Each panel shows a histogram of abundances for a single species (A) or a single variable metabolite (B) across all 61 simulation runs in the 10-species dataset. (C) Nonmetric multidimensional scaling plots of species composition across the 61 original simulation runs, using Bray-Curtis dissimilarity. The distinctive structure in the data arises due to the approach that we used to select initial species compositions, which allowed us to systematically explore the composition space by examining the effects of perturbing individual species. This structure is similar to compositions observed in ordination plots in previous studies (see, for example, references 93 and 94). (D) Principal-component analysis of metabolite concentrations across the 61 original simulation runs. (E and F) The same plots as those described for panels C and D, but panels E and F include all simulation runs with environmental fluctuations in the nutrient inflow. Download FIG S2, TIF file, 1.2 MB. Copyright © 2019 Noecker et al. 2019 Noecker et al. This content is distributed under the terms of the Creative Commons Attribution 4.0 International license.

Clearly, the model and simulations described above represent gross simplifications of the microbiome’s structure, dynamics, and function. Importantly, however, the simplification is also a strength. Specifically, the data obtained from these simulations provide a unique opportunity to examine the relationship between community dynamics and metabolic activity in a tractable model of community metabolism where complete information about the activity and fluxes of each microbial species is available (Fig. S3). Indeed, our multispecies model captures many of the intricacies of bacterial genome-scale metabolism and the interconnectedness (both within and between species) of multiple metabolic processes and yet does so without the additional complexities inherent in *in vivo* communities. Furthermore, in our initial set of simulations, variation in the concentrations of environmental metabolites resulted exclusively from microbial metabolic activity, with no variation in nutrient inflow or other nonmicrobial sources, providing a controlled setting for evaluating the relationship between community members and metabolite concentrations.

10.1128/mSystems.00579-19.4 FIG S3 Cumulative uptake and secretion fluxes for all species and all metabolites, across all 61 simulations. For all analyzed metabolites, the lower panel shows the total cumulative level of secretion or uptake of that metabolite by each species across all 61 simulation runs. The upper panel shows the corresponding environmental concentration at the final time point. Each plot shows fluxes for a single metabolite, with those found in the nutrient inflow shown in the upper section and microbially produced metabolites shown below. Metabolites are ordered by their total variance. Simulations are ordered on the *x* axis as described for Fig. 1 and 2. Download FIG S3, TIF file, 2.9 MB. Copyright © 2019 Noecker et al. 2019 Noecker et al. This content is distributed under the terms of the Creative Commons Attribution 4.0 International license.

### Metabolite variation is driven by diverse microbial mechanisms.

Given the simulated data set described above (for which uptake and secretion fluxes are known), we applied our contribution framework to calculate the contribution of each species to the variation observed in each of the 52 variable metabolites (Fig. S4). The resulting contribution values can be used as ground truth information about the link between microbial activity and environmental metabolites.

10.1128/mSystems.00579-19.5FIG S4Variance contribution profiles for all metabolites. Each plot shows contribution values for a single metabolite in the main 10-species dataset, with those found in the nutrient inflow shown in the upper section and microbially produced metabolites shown below. Metabolites are ordered by their total variance. The relative contribution values, ${\hat{c}}_{i}$, are plotted on the *x* axis. Download FIG S4, TIF file, 1.0 MB.Copyright © 2019 Noecker et al.2019Noecker et al.This content is distributed under the terms of the Creative Commons Attribution 4.0 International license.

To highlight the nature and utility of such contribution values, and to demonstrate how metabolic fluxes translate into contribution profiles, we first describe our results for several example metabolites (Fig. 2). Putrescine, an amino acid fermentation product, is an example of the simplest case, in which one microbial species—Escherichia coli—synthesizes a metabolite that is not utilized or modified by other community members. Variations in the environmental concentrations of putrescine were hence fully determined by the level of secretion from E. coli, which is therefore assigned a relative contribution value of 1 (Fig. 2B). Tetradecanoic acid, in contrast, was introduced (at a constant rate) via the nutrient inflow and utilized by the three *Bacteroides* species in the community to various degrees (primarily by B. ovatus and to a slightly lesser extent by B. thetaiotaomicron). The calculated contribution values successfully attributed variations in the environmental concentrations of this metabolite to these three species and correctly captured the differences in the magnitudes of their effects (Fig. 2C). Variations in concentrations of uracil, another metabolite introduced via the nutrient inflow, were mainly driven by large shifts in its uptake by B. ovatus, but this effect was partially masked by E. rectale, which reduced its uptake when B. ovatus’ uptake flux was high and vice versa. Other species also utilized uracil, but at relatively similar levels across samples, with correspondingly little impact on its variation. These patterns were all captured by the contribution profile obtained by our framework, with B. ovatus assigned a high positive contribution, E. rectale assigned an intermediate negative contribution (reflecting its role in compensating for the effects of B. ovatus), and other species assigned relatively negligible contribution values (Fig. 2D). More-complex species-metabolite relationships were also accurately and effectively summarized. Contribution values for acetate, for example, reflected the cross-feeding interactions that underlie variations in its concentrations (Fig. 2E). It was introduced to the shared environment by several species (primarily Cenarchaeum symbiosum), but most of its variation ultimately depended on the level of uptake by E. rectale. Finally, the contribution profile of succinate demonstrates how extremely strong interspecies interactions can produce contribution values much greater than the observed variance (Fig. 2F). In the simulated data, this metabolite was synthesized by Blautia hydrogenotrophica but was almost always fully utilized by other community members. The contribution calculations suggest that if the synthesis of succinate by B. hydrogenotrophica had not been offset by uptake from other species, the variance in succinate concentration across samples would have been 71.7 times higher than that actually observed. (Note that the difference between positive and negative is always 1.)

Examining the complete set of variable metabolites and calculated contribution values revealed similar patterns of interactions (Fig. S4). Specifically, as for the metabolites discussed above, negative contributions and/or contribution values greater than 1 were widespread. Nearly all metabolites (50 of 52) had at least one species with a negative contribution value, and 36 had at least one species with a contribution value greater than 1. Of the 32 other metabolites with negative contributions, 29 were present in the nutrient inflow and their negative contributions resulted from competition between species for their uptake. This prevalence of negative and extreme values suggests that strong negative interspecies interactions have substantial impacts on metabolite concentrations and that an observed variation in a given metabolite’s concentration often represents the complex outcome of multiple species generating and offsetting much higher variation.

Note also that while the average metabolic uptake/secretion flux of each species and the magnitude of its contribution to the concentration of a given metabolite were generally significantly correlated (Spearman, *P < *0.01 for 49 of the 52 metabolites), the species with the highest flux was often not the largest contributor to variation (26 of the 52 metabolites). Similarly, the variance in a species’ flux was significantly correlated with its contribution for 48 of the metabolites, but for 9 metabolites the species with the most variable flux was still not the largest contributor (due to differences in whether the variable flux generated by one species was compensated by variation in the flux of another). These findings suggest that even if the magnitude and variation of species uptake and secretion fluxes across a set of microbiome samples are known (rather than just the abundances of species, which is the only measure usually assayed), metabolic interdependence between species could still make true contributor species challenging to identify.

Combined, the observations described above highlight the complex relationship between species activity and measured metabolite concentrations, demonstrating the important role of both direct and indirect species interactions.

### Correlation analysis is limited in its ability to detect true microbial contributors to metabolite variation.

Given our observations described above, we next set out to comprehensively assess how accurately pairwise correlation analysis (commonly used for analyzing microbiome-metabolome data) can detect true taxonomic contributors to metabolite variance in this data set. Following numerous microbiome-metabolome studies (17, 28, 30, 34), we considered identifying species-metabolite relationships as a classification task, aiming to identify for each metabolite the set of species that are primarily responsible for the variation observed in its concentration across samples. To this end, true key contributor species for each metabolite were defined as those with a contribution value representing greater than 10% of the total positive contribution values, resulting in a set of 83 species-metabolite key contribution links. On average, each metabolite had only 1.6 key contributors (Fig. S5), even though 7.5 species on average had utilized or synthesized each metabolite at any point. A total of 31.3% of key contributions occurred via synthesis reactions, 66.3% via utilization, and 2.4% (2 instances) via both processes. To mimic a typical microbiome-metabolome correlation analysis, we then calculated the Spearman rank correlations between species abundances and metabolite concentrations across samples and used a *P* value threshold of 0.01 to define significant correlation between species and metabolites. This produced a set of 191 significant species-metabolite correlations, representing putative species-metabolite links. Several examples of these species-metabolite abundance relationships are shown in Fig. S6.

**FIG 3:**
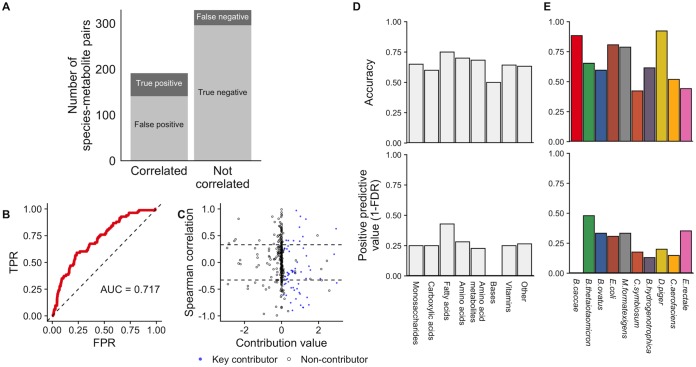
Species-metabolite correlations poorly predict species contributions to metabolite variation. (A) The number of species-metabolite pairs that were significantly correlated (left bar) or not correlated (right bar) and its correspondence with true species-metabolite key contributors (indicated by gray shading). (B) Receiver operating characteristic (ROC) plot, showing the ability of absolute Spearman correlation values to classify key contributors among all species-metabolite pairs. FPR, false-positive rate; TPR, true positive rate. (C) Scatter plot of species-metabolite pairs, showing the poor correspondence between true contribution values (*x* axis) and Spearman correlation (*y* axis). Key contributors are plotted as blue points and others as hollow circles. Dashed lines show significant correlations (*P < *0.01). Species-metabolite pairs with a contribution value greater than 3 in magnitude whose values are not shown. (D and E) Accuracy and positive predictive value of Spearman correlation analysis for detecting true key contributors across metabolite classes (D) and for each of the 10 species (E).

**FIG 4:**
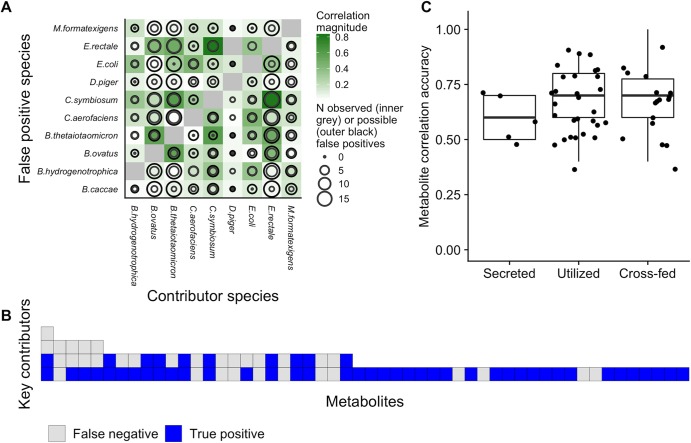
Metabolite and species properties explain correlation-contribution discrepancies. (A) Strongly correlated species pairs produced greater numbers of false-positive metabolite correlations. In the plot, the color of each tile indicates the strength of correlation in the abundances of each pair of species. The size of the outer black circle in each cell represents the number of metabolites for which the species indicated on the *x* axis is a key contributor and the species indicated on the *y* axis is not. The size of the inner circle represents the share of those metabolites for which a false positive was observed for the species on the *y* axis. It can be seen that many false-positive correlations involve the taxa with the strongest interspecies associations: E. rectale, B. ovatus, and B. thetaiotaomicron. (B) Metabolites with more microbial key contributors were more prone to false-negative correlations. Each column represents an analyzed metabolite, ordered by its number of key microbial contributors, which are represented by each tile. The tiles are coded by the correlation outcome for each contributor. (C) Correlations detected key contributors equally accurately regardless of whether a metabolite is secreted, utilized, or cross-fed by the species. Each point represents the accuracy of correlations for a single metabolite across its comparisons with all 10 species.

10.1128/mSystems.00579-19.6 FIG S5 Key contributors and key players driving metabolite variance have similar properties and correlation results. (A) Histograms of the number of key contributor species and key player species for all 52 analyzed metabolites. (B) Number of key contributor and key player relationships for each species. Full species names can be found in Fig. 2. (C to G) Correlation results for identification of key players, as shown in Fig. 3 and 4 for key contributors. (C) The number of species-metabolite pairs that were significantly correlated (left bar) or not correlated (right bar) and correspondence of the number to true species-metabolite key players. (D) Receiver operating characteristic (ROC) plot, showing the ability of absolute Spearman correlation values to classify key players among all species-metabolite pairs. (E and F) Accuracy and positive predictive value of Spearman correlation analysis for detecting true key players across metabolite classes (E) and for each of the 10 species (F). (G) As described for Fig. 4, correlation-based analyses detected key players equally accurately regardless of whether a metabolite was secreted, utilized, or cross-fed by the species. Each point represents the accuracy of correlation-based analysis for a single metabolite across its comparisons with all 10 species. Download FIG S5, TIF file, 1.0 MB. Copyright © 2019 Noecker et al. 2019 Noecker et al. This content is distributed under the terms of the Creative Commons Attribution 4.0 International license.

10.1128/mSystems.00579-19.7 FIG S6 Examples of species-metabolite correlation outcomes in the main 10-species dataset. Each panel plots the concentration of one of the example metabolites shown in Fig. 2 against the abundance of a key contributor or non-contributor species, with annotations of the corresponding Spearman correlation and contribution values. Download FIG S6, TIF file, 0.5 MB. Copyright © 2019 Noecker et al. 2019 Noecker et al. This content is distributed under the terms of the Creative Commons Attribution 4.0 International license.

Comparing this set of significant species-metabolite correlations to the set of species-metabolite key contributors clearly illustrated the difficulty of using univariate associations to infer mechanistic contributions (Fig. 3). Indeed, of the 191 significant species-metabolite correlations, the vast majority (141) were false positives (corresponding to a positive predictive value of only 26.2%) and did not represent true contributor relationships (Fig. 3A). Moreover, more than a third (51 of 141) of these false-positive species-metabolite pairs had no mechanistic connection; i.e., the species did not ever use or produce the metabolite with which it was correlated. Furthermore, for 12 variable metabolites (of 52), none of the key contributors were successfully detected by a correlation analysis. The overall accuracy was somewhat higher (66.5%), reflecting the high number of non-contributors that were also not correlated. Using a stricter cutoff (*P < *0.0001, equivalent to a Bonferroni-corrected value of 0.05) improved the positive predictive value only to 33% and the accuracy only to 77.1%. Indeed, a receiver operating characteristic (ROC) curve analysis (Fig. 3B) produced an area under the curve (AUC) value of 0.72, and overall correlations and scaled contribution values were only weakly associated (Fig. 3C), suggesting that the impact of these findings can be mitigated only partially by changing classification thresholds.

Notably, metabolites of different classes had generally similar correspondences between correlations and contributions (Fig. 3D). Similarly, key contributors corresponding to purely microbially produced metabolites were not identified more accurately than those corresponding to metabolites in the nutrient inflow (66% versus 67%), which is perhaps not surprising since we used a constant inflow across samples (but see also our analysis below with variable inflow). Moreover, the total variance in a metabolite was not associated with the accuracy or predictive value for that metabolite (Spearman rho, *P > *0.1). Across species, contributions were identified most accurately for Desulfovibrio piger, which had a relatively low number of contributions (Fig. 3E; see also Fig. S5C), but the positive predictive value was nonetheless <50% for all species.

Importantly, we additionally confirmed that our findings do not represent an artifact of various specific aspects of our simulation and analysis frameworks, obtaining similar results across several variants (Text S1) (Fig. S5 and S7). First, we evaluated the use of an alternative classification task, aiming to detect all microbes that affect variation in a given metabolite across samples regardless of whether their effects are ultimately reflected in the observed concentrations (i.e., those with large positive or negative contributions), resulting in similar findings (Text S1) (Fig. S5). To assess the impact of dynamic shifts over the duration of each simulation, we also calculated an alternative set of contribution values based on the net steady-state metabolite flux rates at the final time point of each simulation, finding again results that were extremely similar to those determined for the contributions to cumulative variations in concentrations (Text S1). Similarly, we profiled the effects of model simulation parameters on correlation results, including the simulation length and the maximum enzymatic rate *V*_max_, yet again finding minimal effects on contribution and correlation results (Text S1) (Fig. S7).

10.1128/mSystems.00579-19.8 FIG S7 Effects of simulation duration and *V*_max_ parameter on contribution profiles and correlation efficacy. (A) Species abundances after 10-species simulation runs of increasing length. Data from longer simulations increasingly converge towards a consistent profile, as the nutrient medium selects for a growth-optimized composition of species. (B) Metabolite variance decreases with increasing simulation duration. Each line represents the total variance in the concentration of a metabolite across the 61 simulations. The *y* axis is plotted on a log_10_ scale, and the duration of 144 h described in the main results is indicated with a dotted line. (C) Each line represents the number of key contributions by each species across simulation datasets of increasing duration. The total number of key contributors decreases with increasing length. The duration of 144 h described in the main Results section is indicated with a dotted line. (D) Bar plots of correlation and contribution outcomes with increasing simulation duration, with the “C”-labeled bar indicating the number of correlated species-metabolite pairs and the “N” indicating the number of noncorrelated pairs. Datasets generated from longer simulations display more significant correlations and fewer key contributors. The duration of 144 h described in the main Results section is indicated with an asterisk. (E) Overall shifts in prediction metrics for correlation analysis with increasing simulation duration. AUC and predictive values are largely constant. (F) Species compositions generated using different values of the parameter are nearly identical. The color legend is the same as for panel A. (G) Bar plots of correlation and contribution outcomes from simulations with various values of *V*_max_, with the “C”-labeled bar indicating the number of correlated species-metabolite pairs and the “N” indicating the number of noncorrelated pairs. (H) Overall prediction metrics for correlation analysis are largely constant across simulations generated with different *V*_max_ values. Download FIG S7, TIF file, 2.3 MB. Copyright © 2019 Noecker et al. 2019 Noecker et al. This content is distributed under the terms of the Creative Commons Attribution 4.0 International license.

Finally, since our data set is highly structured by the pattern of initial species abundances, we also performed a stratified correlation analysis across groups of samples to confirm that our findings cannot be explained by this variation structure alone. Specifically, we classified samples into subgroups based on the most abundant initial species and calculated species-metabolite correlations within each such subgroup. Inspired by the approach used in a recent large-scale microbiome-metabolome study (36), we considered a species-metabolite association to represent a confirmed link if it was both significant (at a cutoff with the same false-discovery rate [FDR] as applied previously) and in a consistent direction across all sample subgroups. Using this strict classification, only 2 species-metabolite pairs were identified as confirmed links, and only 1 of the 2 pairs represented a key contributor. Similarly, 11 associations were consistent across at least 9 of the 10 subgroups, among which 4 pairs were true key contributors. These observations suggest that such a cross-group strategy could potentially improve predictive value to some extent but would do so at the cost of a substantial decrease in sensitivity.

### Accuracy of correlation-based analysis is species and metabolite specific.

Our analysis described above demonstrated that correlations between species abundances and metabolite concentrations can be poorly associated with the true contribution of species to metabolite variation. We therefore next investigated the origins of such discrepancies. We specifically examined whether individual metabolites or species are predisposed to produce a significant species-metabolite correlation when the species in fact does not contribute to that metabolite variation (i.e., false positives) or to mask such correlation when the species does in fact contribute to this metabolite variation (i.e., false negatives) and, if so, what species and metabolite properties are linked to those outcomes.

To determine whether the identity of the species or metabolite in question is associated with inaccurate identifications of key contributors, we used a regression-based analysis. Specifically, we considered all species-metabolite non-contributor pairs, and fitted a logistic regression model to predict whether a species-metabolite pair exhibited significant correlation (false positive), based on species identities or on metabolite identities or both (see Materials and Methods). We then compared these three models using a likelihood ratio test (LRT) to assess whether species and/or metabolite identities were informative. We similarly considered all species-metabolite key contributor pairs separately, again fitting a logistic regression model based on species identities or on metabolite identities or both to predict whether a pair failed to exhibit significant correlation (false negative).

For non-contributors, we found that false positives were able to be explained largely by species identity (LRT for inclusion of species terms, *P < *10^−13^). Incorporating both species and metabolite identities did not significantly improve the model (LRT for metabolite terms, *P = *0.72). This finding suggests that false positives—i.e., correlations observed between species and the metabolites to which they in fact did not contribute—represent the outcome of interactions at the species level, regardless of the identity of the metabolite in question. This impact of strong interactions between data set features on association test results has been described extensively for other data types (40, 41). Indeed, examining the 141 false positives identified above, we found that many can be explained by the relationships among the three dominant species in this community: E. rectale, B. thetaiotaomicron, and B. ovatus. These species competed strongly for carbon sources (and utilized their maximum allocation of sucrose, glucose, and fructose at nearly every step of the simulation), and their abundances were therefore negatively correlated. As a result, metabolites whose concentrations varied due to the activity of one of these species were also frequently correlated with the results seen with the other two. In total, 32 false-positive correlations paired one of these species with a metabolite for which another species in this trio was a key contributor. More generally, we found that the probability of a false-positive correlation for a particular species and metabolite depended on the species’ correlation with the true key contributors for that metabolite (*P = *0.006, Spearman rho between share of false positives and interspecies correlation; Fig. 4A). Moreover, the maximum correlation that each species showed with any other species is a strong predictor of its overall specificity, which ranges widely from 33.3% for E. rectale to 92% for D. piger (Spearman rho = −0.84, *P* *= *0.002). Species identity was also similarly predictive of whether a significantly correlated metabolite-species pair represented a true contributor versus a false positive (Text S1).

In the case of key contributors, we found that false-negative correlations can be explained largely by metabolite identity (LRT for metabolite terms, *P = *0.002, although the species involved was also somewhat informative with LRT *P = *0.08). Put differently, a lack of correlation between the abundance of a key contributor species and the concentration of the metabolite to which it contributed was determined mainly by the nature of the metabolite in question. This lack of correlation between a given metabolite and its contributors could have resulted from competition or exchange of a metabolite between multiple species, such that none of the involved species end up strongly associated with the final outcome on their own. Indeed, across all metabolites, the average correlation between a metabolite and its key contributors was found to be negatively associated with its number of key contributors (Spearman rho = −0.45, *P = *0.0008). The number of key contributors for any metabolite was also thus negatively associated with the sensitivity of contributor detection for that metabolite (Spearman rho = −0.48, *P = *0.0004; Fig. 4B). We further hypothesized that false-negative outcomes might be more common for metabolites with more or larger negative species contributions, since these, by definition, mask or compensate for the activity of key contributor species. While all metabolites with a false-negative outcome did correspond to at least one species with a negative contribution value, as mentioned above, this was true for nearly all analyzed metabolites (50/52), and the number of negative contributing species was not associated with the occurrence of a false-negative correlation (*P = *0.86, Wilcoxon rank sum test). Moreover, we also did not observe any effect of the average concentration of a metabolite on the sensitivity and accuracy of its detection via correlation analysis or of whether it was secreted, utilized, or cross-fed (Fig. 4C). In summary, our analysis suggests that the most informative factor in determining whether a metabolite’s key contributor can be detected by a correlation analysis is simply whether there are other community members (key contributors) that also impact the observed concentration of that metabolite.

### Environmental fluctuations in metabolite concentrations impact detection of key contributors.

Our analyses described above all focused on a single simulated data set in which the nutrient inflow was constant across all samples, meaning that metabolite variation was fully governed by microbial activity. However, in reality, metabolite variation can and does arise also from nonmicrobial sources, potentially affecting both the landscape of key microbial contributors and our ability to detect them via correlation-based analyses. To explore the impact of environmental fluctuations, we therefore ran several sets of additional simulations with various degrees of nutrient fluctuation, designed to emulate a range of levels of stochastic variability in nutrient availability across the simulated mouse gut communities, which could arise naturally due to, for example, lot-to-lot variability in mouse chow and/or small variations in intestinal physiology between mice. In these simulations, we maintained the same set of 61 initial species compositions but introduced small random adjustments to the nutrient inflow, sampling inflow concentrations for each compound in each simulation from a normal distribution with a mean equal to the compound’s original inflow rate and a standard deviation ranging from 0.5% to 10% of the mean in 8 increments (see Materials and Methods). For each of the resulting 8 data sets, we again calculated contribution values (with the added element of the nutrient inflow as a potential contributor to variance), identified key contributors, and compared them with the results of a correlation analysis.

**FIG 5:**
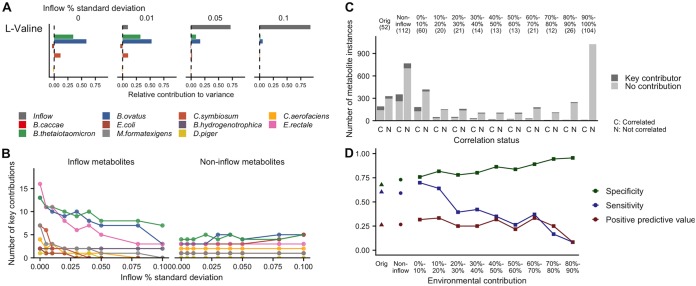
Environmental fluctuations impact correlation-contributor sensitivity and specificity. (A) Example set of contribution profiles for a single inflow metabolite, l-valine, with increasing fluctuations in its inflow. The relative contribution values for each species and for the inflow are shown for 4 sets of simulation runs, each with a different degree of fluctuation. The label on each plot indicates the relative standard deviation (coefficient of variation) of inflow metabolite concentrations for that set of simulations. The microbial contributions to variance in l-valine concentrations became relatively smaller with increasing variation from the external environment. (B) Shifts in key microbial contributors with increasing environmental inflow fluctuations. The number of key contributions of each species (represented by the same colors as in panel A) to the 52 analyzed metabolites is shown separately for metabolites present in and absent from the nutrient inflow. Levels of microbial contributors to inflow metabolites decreased as environmental contributions increased, but this effect differed between taxa. (C) Correlation analysis failed to detect key microbial contributors regardless of the size of contribution from external inflow variation. Across all sets of simulations, metabolites were binned based on the percentage of total positive contribution from the external inflow. The bar plots shown have the same format as that presented in Fig. 3A, showing the number of species-metabolite pairs that were significantly correlated (left bar) or not significantly correlated (right bar) and its correspondence with true species-metabolite key contributors (indicated by shade of gray). The first two bars (labeled “Orig”) represent the original set of simulations (replicating Fig. 3A). The next two represent results from metabolites not present in the inflow across all levels of inflow fluctuations. The remaining bars represent results from metabolites with increasing levels of environmental contribution. (D) Correlation analysis detected key microbial contributors with increased specificity, decreased sensitivity, and generally consistent positive predictive value with increasing contribution from the external inflow. Sensitivity, specificity, and positive predictive values are shown for same environmental contribution bins as those described for panel C.

Examining the obtained contribution values, we found, as expected, that variation in inflow quantities can outweigh the variation in microbial fluxes and that as the variation in inflow increased, its contribution to metabolite variation increased at the expense of the contributions of community members (Fig. 5A). As a result, the number of key contributions attributed to each species decreased for metabolites in the nutrient inflow (Fig. 5B). Interestingly, however, some species lost their contributions more gradually than others and in some cases even became key contributors for additional metabolites. For most metabolites, the top microbial contributor did not change with increasing fluctuations (Text S1).

We next examined how correlation-based detection of key microbial contributors was affected by these inflow fluctuations. We assigned each of the 52 metabolites in each of the 9 data sets (the original data set with no inflow fluctuations and the 8 data sets with various degrees of fluctuations) to bins according to the level of contribution attributed to the inflow for this metabolite at that degree of fluctuation (see Materials and Methods). We then evaluated the performance of correlation analysis for each bin separately. The share of true key contributors naturally decreased rapidly with increasing environmental contribution, as did the number of significantly correlated species-metabolite pairs (Fig. 5C). Importantly, however, the sensitivity of correlations decreased substantially with the level of contribution attributed to the inflow, but the specificity in fact increased from 67.7% to 92.3% (Fig. 5D). This suggests that while environmental fluctuations disrupted the signal linking microbial species with the metabolites that they impacted, they also disrupted indirect associations between species and metabolites (false positives). Overall, however, the AUC did not change significantly with increasing environmental contribution (Fig. S8A), and the positive predictive value was similarly relatively stable (and was never higher than 37%). Interestingly, the detection of some metabolites not present in the inflow was also affected by inflow fluctuations in a similar manner (Text S1) (Fig. S8B).

10.1128/mSystems.00579-19.9 FIG S8 (A) Minimal effects of environmental fluctuations on overall correlation performance. ROC curves are shown for sets of metabolites with increasing environmental contribution. None of the levels of environmental contribution had significantly different areas under the curve, based on 95% confidence intervals calculated using bootstrap resampling with 500 replicates. (B) The sensitivity and specificity of correlation analysis to detect key microbial contributors to metabolites not present in the inflow are affected by variation in metabolic inflow. Each point represents the specificity, sensitivity, or positive predictive value determined for the 14 analyzed metabolites not present in the inflow in a dataset of 61 simulations. Percent standard deviation (coefficient of variation) in inflow metabolite concentrations for each set of simulations is plotted on the *x* axis. Download FIG S8, TIF file, 0.3 MB. Copyright © 2019 Noecker et al. 2019 Noecker et al. This content is distributed under the terms of the Creative Commons Attribution 4.0 International license.

### Correlation analysis is similarly limited in accuracy in simulations of the more complex and diverse human gut microbiota.

**FIG 6:**
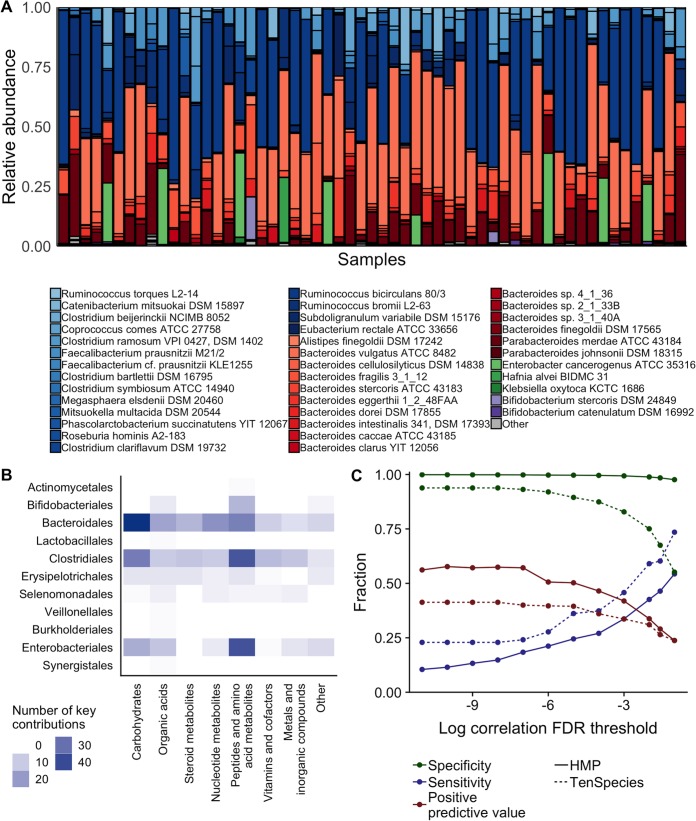
Correlation-contribution discrepancies persist in simulations of complex human gut-based microbiota. (A) Species abundances of the 57 Human Microbiome Project (HMP)-based simulations at the 144-h time point. Species are indicated as follows: phylum *Firmicutes*, shades of blue; *Bacteroidetes*, red; *Proteobacteria*, green; *Actinobacteria*, purple. (B) Key contributions to metabolite variation across the HMP-based data set, summarized at the level of taxonomic orders and metabolite categories. (C) Performance of correlation analysis for identifying key species-metabolite contributors in the HMP-based data set (solid lines) compared with the original 10-species data set (dashed lines) across various significance levels, using Benjamini-Hochberg false-discovery-rate (FDR)-corrected *P* values.

Our results have illustrated consistent discrepancies between microbe-metabolite correlations and microbial contributions to metabolite variation in model 10-species communities. However, it is unclear to what extent these discrepancies were influenced by the simple species compositions and structure variance of the previous data set. Here, we therefore examined whether these findings generalize to the more complex mammalian gut communities, which often include many times more taxa and a more uneven distribution across individuals. To do so, we ran an additional set of simulations emulating human gut microbiota transplanted into gnotobiotic mice. We first mapped 16S rRNA sequence variants from the Human Microbiome Project (HMP) (61) to the genomes of the AGORA model collection at 97% sequence identity (48) and selected the 57 samples for which greater than 25% of the total abundance of sequence variants were mapped to an AGORA model. The total share of mapped reads averaged 36.7% across these samples, with a maximum of 73.5%. Despite the variations in mapping rates, the obtained species distribution of the mapped reads displayed properties typical of Western gut microbiomes, including a predominance of *Bacteroidetes* and *Firmicutes* phyla along with various lower abundances of *Actinobacteria* and *Proteobacteria* (Fig. 6A). The number of species identified in each sample ranged from 23 to 62, with a median of 42. We ran simulations based on each sample by setting the initial species relative abundances according to the relative abundances of mapped reads while maintaining the same physical parameters as those used in the previous simulations (see Materials and Methods for additional details). We used nutrient inflow quantities with 1% standard deviation between samples. Initial species compositions shifted in abundance in consistent ways over the simulation time course, as they tended to become dominated by a limited number of fast-growing species (Fig. S9A). The obtained metabolite profiles were highly variable, with a median coefficient of variation of 71% across 222 metabolites (Fig. S9B). As described above, we calculated contribution values for this data set, identifying true species-metabolite contributions. Overall, in this data set, a smaller share of the possible species-metabolite pairs were identified as true contributors (392 of 29,082 possible pairs), and only 35.1% of the species (46 of 131) were identified as key contributors to any metabolite. The genera with the most contributions were *Bacteroides*, *Ruminococcus*, and *Enterobacter*, which also represented three of the four most abundant genera in the final data set (Fig. 6B).

10.1128/mSystems.00579-19.10 FIG S9 (A) Progression of HMP-based simulations. A principal-coordinate analysis of the species compositions of the 57 HMP-based simulations at their initial and final time points was performed using the Bray-Curtis dissimilarity metric. Initial compositions tended to become dominated by a limited number of fast-growing species, leading to distinct subgroups. (B) Metabolite variation across HMP-based simulations. A principal-component analysis of the metabolite concentration data at the final simulation time point was performed. (C to F) Examples of species-metabolite correlation outcomes in the HMP-based dataset. Each panel plots the concentration of an example metabolite against the abundance of a key contributor or non-contributor species, with annotations of the corresponding Spearman correlation and contribution values. (G) The specificity of species-metabolite correlation analysis is associated with species prevalence. Each point represents a species that provided at least one key contribution to metabolite variation. The *x* axis categorizes species into quartiles based on the number of samples in which they appear. Species that are present in a wider subset of the dataset have a higher rate of false-positive correlations (lower specificity). (H) The sensitivity of species-metabolite correlation analysis is related to metabolite class and cross-feeding status. Green bars represent the overall sensitivity of identification of key contributor species-metabolite pairs within that category. Orange bars represent the share of metabolites in that category that are both synthesized and utilized by community members (cross-fed). Download FIG S9, TIF file, 0.9 MB. Copyright © 2019 Noecker et al. 2019 Noecker et al. This content is distributed under the terms of the Creative Commons Attribution 4.0 International license.

We again performed a species-metabolite pairwise Spearman correlation analysis and compared the results with the set of true contributors (full data and results are shown for several example species-metabolite pairs in Fig. S9C to F). Since a smaller share of species-metabolite pairs were significantly correlated in this noisier data set, and in order to fairly perform comparisons with the previous data set while accounting for the larger number of hypothesis tests, we defined correlation significance based on an equivalent Benjamini-Hochberg estimated false-discovery rate (0.027) as the *P* value cutoff of <0.01 used for the previous data set. Among the species-metabolite pairs, 2.2% displayed significant correlations at this cutoff (*P < *0.0006). This level of correlation is comparable to that seen in a recent microbiome-metabolome study of the colon of healthy humans (51), in which 1.4% of operational taxonomic unit (OTU)-metabolite pairs displayed Spearman correlation coefficients of the same effect size. In our data set, correlation analysis detected contributors with high specificity (98.4%) and an area under the ROC curve of 0.89, mostly owing to the very high number of species-metabolite pairs that did not represent true contributions. However, the positive predictive value was still only 29.0% at this cutoff, rising to as high as 57% when using a stricter *P* value cutoff of <10^−10^. We compared these classification results with those determined for the original data set, finding that despite the difference in overall AUC, the sensitivity and predictive values in this more diverse data set were similar to or worse than those observed in the 10-species data set at commonly used FDR thresholds between 0.1 and 0.01 (Fig. 6C). Moreover, as in the 10-species data set, a large share of false-positive species-metabolite pairs (65.4%, 291 of 445) also involved species with no capacity to impact the metabolite in question.

Examining factors that underlie discrepancies between correlation-based analysis and true contribution values, we found that the outcomes of correlation analysis were influenced by the same factors as those observed in the model community data set, as well as by several additional characteristics. False-positive classifications were, again, driven by interspecies covariance. Species significantly correlated (at 10% FDR) with a true key contributor for a metabolite were 13.6 times more likely to have a false-positive correlation with that metabolite than species with no such link (*P < *10^−16^). Notably, the false-positive rate determined for a given species was also substantially affected by its prevalence; the number of samples in which a species was present was negatively associated with its specificity (Spearman rho = −0.57, *P = *0.002, Fig. S9G), among species with at least 3 key contributions. In other words, widely prevalent species were more prone to false-positive correlations than rarer species. False-negative contributions were again influenced by properties of both metabolites and species. As seen with the 10-species data set, species contributions to metabolites with two or more key contributors were 5.2 times more likely to not be correlated than those that represented the sole key contribution for a metabolite (*P < *10^−10^, Fisher’s exact test). In this data set, an elevated share of those metabolites with multiple key contributors was cross-fed between different species (*P = *0.00007, Fisher’s exact test) and, correspondingly, key contributors for cross-fed metabolites were also 1.6 times less likely to be significantly correlated (*P = *0.02). Importantly, both cross-feeding and false-negative outcomes were found to occur variably across metabolite classes, with nucleotide metabolites having the highest rates of both phenomena (Fig. S9H). Taken together, our simulations and analyses of this more realistic microbiota simulation demonstrate that correlation analysis can have a somewhat greater utility in a microbial community data set with greater complexity and variability, but that the results still display a high false-discovery rate and are strongly influenced by properties of individual metabolites and species.

## DISCUSSION

### Insights and implications for microbiome-metabolome analyses.

As described above, we investigated the ability of correlation-based analyses to detect key microbial contributors responsible for variation in metabolite concentrations across samples in simulated data sets. Our findings suggest that microbe-metabolite correlation analysis may be a useful approach for exploratory analyses but that such analyses have caveats and can be impacted by several factors. Below, we elaborate on a set of practical conclusions and their implications for the analysis and interpretation of microbiome-metabolome studies. Note, though, that the precise correlation performance statistics observed in our simulated data sets may not fully generalize to diverse real-world microbiome-metabolome data sets; nevertheless, the principles illustrated by these data sets are important to consider in interpreting microbiome-metabolome results.

### Association-based analyses of microbiome-metabolome assays can have low predictive value for detecting direct species-metabolite relationships.

Microbiome-metabolome association studies have been previously proposed to represent a powerful tool for the identification of causal mechanisms of microbiome metabolism (53), and, indeed, such studies often present detected associations as evidence for mechanistic relationships (11, 33, 35–37). However, our analysis suggested that the positive predictive value of significant species-metabolite correlations for identifying true microbial contributors can be extremely low: less than 50% across all settings, as low as 10% in the context of large environmental fluctuations, and 29% in simulations based directly on human gut composition. Although we have not evaluated many variables and data set characteristics that could influence these statistics, these findings are also supported by those of recent experimental studies pairing microbiome-metabolite correlation analysis with *in vitro* monoculture validations, and those studies have similarly anecdotally described many false-positive correlations (36, 39). Additionally, the somewhat low sensitivity observed in our analyses suggests that a lack of association is not necessarily sufficient to justify rejection of a hypothesis suggesting that a particular microbial taxon impacts a particular metabolite. The choice of correlation threshold should therefore be made carefully, taking into account the complexity of the community and the environmental context. In general, identified correlations between microbial taxa and metabolites should be interpreted very conservatively and used mostly to prioritize microbe-metabolite relationships for follow-up validation studies (e.g., via culture-based studies or germfree model organism colonization). This approach has already been applied successfully in some cases (39). Another potential strategy for improving the predictive value of such correlation-based analyses is to examine whether they replicate across multiple conditions (e.g., discovery and validation cohorts [36]). Indeed, we found that a correlation analysis may provide stronger evidence for a contributor relationship if it persists across different contexts or data subsets. This was true across subsets of the original 10-species data set, as well as across our 9 10-species simulated data sets with various environmental fluctuations. In that comparison, the 43 species-metabolite pairs that were consistently significantly correlated in every data set were 2.1 times more likely to denote true key contributor relationships than other significant correlations (Fisher’s exact test, *P = *0.05), although the positive predictive value was still relatively low (39.5%). However, of the limited number of significant correlations shared between our original 10-species and HMP-based data sets (*n = *5), all were false positives in both data sets, again suggesting the need for caution.

### The predictive power of correlation-based analysis is species, metabolite, and context dependent.

In our data sets, metabolites varied widely in both their contribution profiles and their detectability via correlation analysis. In particular, the key contributors for metabolites acted upon by fewer species and, potentially, those that are not exchanged between different species were identified more readily. Moreover, in our simulations of human gut communities, contributions by the less prevalent species were identified much more accurately than those by widely found species, indicating that hypotheses based on associations of rarer species should potentially be prioritized. Correlation analysis may thus identify microbes involved in specialized secondary metabolic processes (e.g., products of complex biosynthetic pathways) performed by rare taxa more readily than those involved in the more widespread processes. Similarly, we found that the species-metabolite correlations for species that are strongly associated with other taxa (e.g., those with tight interactions with other community members) are often spurious, suggesting that such correlations should be regarded less confidently.

### External metabolic fluctuations can strongly impact the detection of microbial contributions.

Our analysis of the impact of environmental fluctuations suggested that the presence of environmental variability corresponding to a diverse set of samples could in fact increase correlation specificity. We also found that the sensitivity of correlation analysis rapidly decreased (from 60% to 9%) with increasing environmental fluctuations. These observations suggest that while a tightly controlled environment (e.g., identical diets) is intuitively expected to increase the strength of microbiome-metabolome studies, its value depends on the study priorities. Specifically, if the goal is to identify clear-cut microbial drivers of health- and disease-associated metabolite shifts, stochastic variation in nutrient availability could be beneficial as it may reduce the rate of false-positive associations. In contrast, for studies searching for a particular microbial taxon’s involvement in a particular process (e.g., aiming to determine whether an ingested probiotic impacts aspects of gut metabolism), a more controlled environment may be favorable. It should, however, be noted that our findings were based on environmental fluctuations that were stochastic, uniform, and independent, conditions which may not hold for many real-life sources of environmental variation such as diet or host circadian rhythms. It is also worth noting that in our simulations, microbial fluxes for some environmental metabolites could be drowned out by as little as 0.5% variation in nutrient inflow quantities, while others still had substantial microbial contributions even with a 10% variation in inflow. In interpreting an observed association, the scale of possible microbial variation relative to external variation should therefore be taken into account.

### Mechanistic reference information can improve the predictive power of microbiome-metabolome studies.

**FIG 7:**
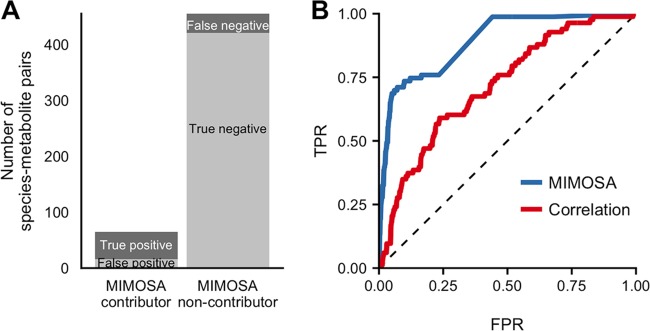
MIMOSA identified key microbial contributors more accurately than correlation analysis. (A) Number of species-metabolite pairs that were identified as potential contributors (left bar) or not (right bar) by MIMOSA and its correspondence with true key contributors. (B) Receiver operating characteristic (ROC) plot, showing the ability of both MIMOSA and absolute Spearman correlation values to classify key contributors among all species-metabolite pairs.

In our simulated data set, 36% of the false-positive correlations occurred between a metabolite and a species that was in fact not capable of taking up or secreting that metabolite. Ruling out such falsely detected links would substantially improve the positive predictive value of a correlation-based analysis. One approach for doing so is that of utilizing genomic information, which can be obtained or predicted for many microbial taxa (62). By coupling such genomic information with metabolic databases such as KEGG (Kyoto Encyclopedia of Genes and Genomes) or MetaCyc (63, 64), researchers can filter out correlation-based links that likely do not represent feasible causative relationships. Further improvement can be obtained by integrating such reference information directly into the analysis. Indeed, we previously introduced a computational framework, termed MIMOSA (model-based integration of metabolite observations and species abundances) (65), that utilizes a simple community-wide metabolic model to assess whether measured metabolite variation is consistent with shifts in community metabolic potential and to identify potential contributing taxa. MIMOSA has been applied to various host-associated microbiomes from various body sites and from human and mouse hosts (12, 66–68). Applying MIMOSA to the simulated species-metabolite data set analyzed above (see Materials and Methods), we found that it indeed identified key contributors significantly more accurately than a correlation-based analysis, with an AUC of 0.89 (Fig. 7). Notably, in this analysis, we assumed MIMOSA had access to the correct set of metabolic reactions possessed by each species. Using standard less-complete information obtained directly from the KEGG database (as done regularly when using this tool) reduced the number of metabolites that could be analyzed from 52 to 39, with improved specificity (96%) and positive predictive value (61%) and an ultimately comparable AUC (0.74). Combined, these findings suggest that reference model-based approaches can provide stronger evidence for mechanistic relationships than strictly correlation-based methods but that their use depends on complete and high-quality metabolic reference databases.

### Future opportunities and challenges.

Microbiome-metabolome studies have an important role in microbial ecology research. They specifically have great potential to dissect the metabolic interactions of complex microbial communities and to unify “top down” and “bottom up” microbiome research approaches by providing mechanistic information at a systems level. Moreover, from a translational perspective, microbiome-metabolome studies can inform efforts to design targeted therapies to alter specific microbial or metabolic features of a community (13). Such interventions require first identifying putative targets, which in many cases may entail identifying the key contributor species that drive observed shifts in a particular beneficial or detrimental metabolic phenotype.

Importantly, while we show here that a correlation-based analysis may be limited in its ability to identify these key microbe-metabolite links, this does not necessarily imply an inherent limitation of microbiome-metabolome data. For example, analyzing our HMP-based data set, we found that species abundance is in fact a very good proxy for metabolic activity (median Pearson correlation of 0.996 between abundance and flux for all species-metabolite pairs), meaning that the variance in total species abundance drastically outweighed the individual-level variance in flux rates. Further examining whether the false-negative associations in our original data set stemmed from a disconnect between the abundance of a species and its metabolite uptake or secretion rates, we identified only 2 undetected key contributor pairs that could be explained by such a discrepancy. This analysis suggests that taxonomic-abundance data can be sufficient to explain and model community metabolic variation to a great extent, despite common concerns about potential discrepancies between community composition and function. It also suggests that metatranscriptomic expression data may not provide much additional value for this purpose, as other studies have indicated previously (62, 69, 70).

Given the increasing prevalence of microbiome-metabolome studies, their promise, and the caveats concerning association-based research discussed above, further development of computational and statistical methods for analyzing such data sets is clearly needed. Possible directions include the use of multispecies dynamic metabolic models that can replicate experimental observations (71), multivariate approaches for deconvolving interactions between species and the environment (72, 73), and probabilistic methods that can integrate prior information while accommodating other unknown mechanisms (38, 74). The analytical framework for calculating taxon-metabolite contributions and the use of dynamic simulations demonstrated here can inform both the future development and the evaluation of such methods.

There is a continuing need for gold standards and evaluation of methods in microbiome-metabolome analysis, as this report represents only a first step. We focused specifically on one type of research issue: identification of microbial taxa directly responsible for variation in metabolite concentrations between samples in a cross-sectional study design. Although this focus describes many recent microbiome-metabolome studies, other studies may address a wide range of complementary research issues and, correspondingly, the desired “ground truth” can take different forms. Depending on the objective, an alternative definition of a taxon-metabolite relationship may be required. For example, it may be valuable to identify key contributors that act via alternative mechanisms, such as by modifying substrate availability or environmental conditions, or to distinguish metabolite variation arising in response to a perturbation from variation due to differences in steady-state metabolism between communities. Additionally, we have not evaluated the ability of microbe-metabolite correlation studies to detect the effects of environmental metabolites (including, e.g., antibiotics) on specific microbial community members.

It is also worth noting several important limitations of our study framework. First and foremost, our findings rely purely on an *in silico* system that may not capture many aspects of community ecology and metabolism. For example, it is possible that the predictive value of correlation analysis, as well as of other analytical methods, in this system differs substantially from that in true biological systems. Our rationale for using an *in silico* framework is discussed above, but we hope that future evaluation analyses will take advantage of ongoing technology developments in mass spectrometry and stable isotope probing to define key microbial contributors based on experimental, quantitative, species-specific community flux data (75–77). Such evaluations can also make use of data sets comparing community microbiome-metabolome data with *in vitro* monoculture or monocolonization data (39, 43, 44). Our study also considered only a small number of data sets whose specific configurations might not be representative of the levels of variation that occur in typical microbiome studies. In particular, the 10-species data set describes a simple set of communities with high between-sample similarity and distinctive compositional structure, which can affect the observed relationships between correlation and contribution values. Observing such a variance structure in an experimental data set may reflect the effect of some key experimental variables (such as sampling site or pH) and may prompt researchers to avoid using correlation analysis altogether or to avoid considering such variables as confounding factors when calculating microbe-metabolite correlations (see, for example, reference 36). Indeed, an important future direction would be to evaluate microbiome-metabolome analysis methods in collections of microbiomes spanning healthy and disease states or that are influenced by other confounding environmental variables. Many other relevant community and data set properties could also affect the outcomes of microbiome-metabolome analysis, including community diversity and stability, sample size, and measurement error. Another important consideration that could affect our findings is sampling time. Indeed, in our study, we ran simulations for a long and yet limited duration, which might have resulted in compositions that differed from those present in real systems in a steady state. Microbiome-metabolome studies should similarly consider whether communities of interest had undergone a recent transition or perturbation versus an being maintained in an extended steady state prior to sampling.

Ultimately, much remains to be learned about the many processes through which complex microbial communities shape their environment. The first major call for the application of metabolomics to microbiome research, published 10 years ago (78), noted that new methods will be necessary to integrate genomic and metabolic data and to inform the prediction of community metabolic properties from metagenomes. Now that microbiome-metabolome data sets are widely available, ongoing development of analysis methods for these studies has great potential to generate new knowledge. Moreover, future work in this area stands to benefit from the utility of dynamic and multiscale metabolic modeling. Detailed mechanistic simulations are used widely in astronomy, climate science, and other fields to make methodological choices and assess possible experimental outcomes under conditions in which ground truth measurements are unavailable or difficult to obtain (79, 80). An analogous strategy may be similarly fruitful in microbiome research.

## MATERIALS AND METHODS

### Derivation of species contributors to variation.

We derived an expression representing the contribution of each species to the variance in the concentration of each metabolite. While we describe this calculation in terms of species, a similar calculation could be done at the level of phyla, strains, or any grouping of the community for which metabolite secretion and uptake fluxes are available.

The concentration of a given metabolite *M* at the end of a single simulation run is a function of the uptake and secretion fluxes (responding to the species’ degradation and synthesis activities) of the *n* species, the environmental inflow over all time steps *m*_in_, and the dilution *m*_out_ of the chemostat over all time steps as follows:

$$M=\overset{n}{\underset{i=1}{\sum }}m_{i}+m_{\text{in}}-m_{\text{out}}$$

The value of *m*_out_ at a given time step *t* is the product of the dilution rate *D* and the metabolite concentration at the previous time point (see above). This fact can be used to express *m*_out_ in terms of all the previously recorded environmental inflow and microbial activities. The metabolite concentration at any time point *t*, *M*(*t*), is calculated by the following equation:

$$M(t)=\overset{t-1}{\underset{\text{k}=1}{\sum }}\left[{\left(1-D\right)}^{t-k-1}\overset{n}{\underset{i=1}{\sum }}m_{\mathrm{ik}}\right]+m_{\mathrm{in}}\overset{t-1}{\underset{k=1}{\sum }}{\left(1-D\right)}^{k}$$

where *m_ik_* represents the activity of species *i* at a single time point *k.* We can then ignore dilution outflow by replacing each activity value *m_i_* in the final concentration calculation shown above with a value corrected for the mitigating effect of chemostat dilution over the course of the simulation up to time *t*, defined here as m_i_*. m_i_* represents the total amount of a compound secreted or imported by species *i* minus the share of that quantity that is eventually diluted out over the course of the simulation as follows:

$$m_{i}^{\text{*}}=\overset{t-1}{\underset{k=1}{\sum }}{\left(1-D\right)}^{t-k-1}m_{\mathrm{ik}}$$

and thus,

$$M=m_{\mathrm{in}}+\overset{n}{\underset{i=1}{\sum }}m_{i}^{\text{*}}$$

In this work, we refer to “environmental fluctuations” as the effect of the independently parameterized nutrient inflow, *m_in_*, and where not otherwise specified we use *m_i_* to imply m_i_*, a species activity quantity that accounts for the corresponding subsequent dilution out of the system.

Using the expression shown above, *var*(*M*) can then be clearly expressed as a sum of correlated environmental and microbial random variables as follows:

$$\begin{array}{c} \text{var}\left(M\right)=\overset{n}{\underset{i=1}{\sum }}\overset{n}{\underset{j=1}{\sum }}\text{cov}\left(m_{i},m_{j}\right)+\overset{n}{\underset{i=1}{\sum }}\text{cov}\left(m_{i},m_{\text{env}}\right)=\overset{n}{\underset{j=1}{\sum }}\text{var}\left(m_{j}\right)+\text{var}\left(m_{\text{env}}\right)+2\overset{n}{\underset{i=1}{\sum }}\overset{n}{\underset{j=i+1}{\sum }}\text{cov}(m_{i},m_{j})+2\overset{n}{\underset{i=1}{\sum }}\text{cov}\left(m_{i},m_{\text{env}}\right) \end{array}$$

This expression can then be partitioned additively into *n + 1* terms representing the contribution of each microbial species and of fluctuations in the environmental nutrient inflow as follows:

$$c_{i}=\overset{n}{\underset{j=1}{\sum }}\text{cov}(m_{i},m_{j})+\text{cov}(m_{i},m_{\text{env}})=\text{var}\left(m_{i}\right)+\underset{j\neq i}{\sum }\text{cov}\left(m_{i},m_{j}\right)+\text{cov}(m_{i},m_{\text{env}})$$

Each contribution value *c_i_* is also equivalent to the covariance of the activity *m_i_* with the total of metabolite concentrations *M*. This equivalence can be seen using the definition of covariance and rearranging the terms above (here assuming no environmental contribution for clarity) as follows:

$$c_{i}=\overset{n}{\underset{j=1}{\sum }}\text{cov}\left(m_{i},m_{j}\right)=\overset{n}{\underset{j=1}{\sum }}E\left\{\left[m_{i}-E\left(m_{i}\right)\right]\left[m_{j}-E\left(m_{j}\right)\right]\right\}=E\left[m_{i}-E\left(m_{i}\right)\right]\overset{n}{\underset{j=1}{\sum }}E\left[m_{j}-E\left(m_{j}\right)\right]=E\left[m_{i}-E\left(m_{i}\right)\right]\text{*}E\left[M-E\left(M\right)\right]=\text{cov}(m_{i},M)$$

### Multispecies dynamic flux balance analysis modeling.

In this study, we simulated the growth and metabolism of a community of 10 representative gut species that was previously explored experimentally (57). We specifically utilized a previously introduced multiscale framework for modeling the dynamics and metabolism of multiple microbial species in a well-mixed shared nutrient environment (51, 52). This framework assumes that the aim of each species in the community is to maximize its own growth on a short time scale given available nutrients and uses flux balance analysis to predict the growth and metabolic activity of each species on this small time scale (56). The shared environment is then iteratively updated based on the species’ predicted growth, uptake, and secretion rates, such that metabolic interactions are mediated via the environment as a natural by-product of species activities rather than being explicitly modeled (81).

We used genome-scale metabolic model reconstructions of the 10 community members from AGORA collection version 1.01 (48), which have been consistently curated to remove or modify thermodynamically unfavorable reactions, remove futile cycles, and confirm growth in anaerobic environments on expected carbon sources, with additional curation for several biosynthesis pathways. COBRA (constraint-based reconstruction and analysis) toolbox version 2.0 was used to convert each AGORA model to MATLAB format (82). The growth and metabolism of the 10-species community were simulated in a chemostat setting in 15-min time intervals. We set the chemostat volume to be approximately equal to that of a mouse gut (0.00134 liter [83]). We similarly set metabolite inflows to emulate the macronutrient and micronutrient quantities in a corn-based mouse chow (57).

The simulations were performed following a previously introduced procedure (52) and were repeated for each time step *t_n_* as follows. First, the maximum rates of uptake for all metabolites by all species, denoted as $v_{\mathrm{jk}}$ for metabolite *j* and species *k*, were calculated based on Michaelis-Menten single-substrate kinetics, with assumed universal values for maximum rate *V*_max_ and transporter affinity *K_m_* for all metabolites. $v_{\mathrm{jk}}$ was further constrained based on an allocation of the metabolite’s environmental concentration to each species in proportion to its biomass. Then, the steady-state reaction fluxes were determined for each species *k* at time point *t_n_* by maximizing the growth rate *μ_k_*, within the obtained constraints on environmental metabolite uptake. To obtain a single and consistent flux solution for each species, the total flux activity for each species (i.e., the sum of absolute fluxes given the predicted optimal growth rate) was minimized, under the assumption that organisms prefer to operate their metabolism with minimal enzymatic cost (84). The optimal flux solutions were solved using linear programming with GLPK (GNU linear programming kit, www.gnu.org/software/glpk). With the resulting flux and growth rate information, the total biomass of each species *k*, bio*_k_*(*t_n_*), was updated for the next time point *t_n+_*_1_, using a standard exponential growth function incorporating dilution as follows:

$${\text{bio}}_{k}(t_{n+1})={\text{bio}}_{k}(t_{n})e^{\mu_{k}\Delta t}-{\text{bio}}_{k}(t_{n})D\Delta t$$

where *D* is the dilution rate. We set *D* to 0.0472 per h in order to obtain community growth rates consistent with the observed average growth rate of the three most abundant species growing under 47 different sets of carbon conditions (85). The total amount of uptake or secretion for each species *k* and metabolite *j* over a single time step was then calculated as previously derived (52) as follows:

$$m_{\text{FBA}}^{\mathrm{jk}}(t_{n})=\frac{v_{\mathrm{jk}}}{{}_{k}}{\text{* bio}}_{k}(t_{n})(e^{{}_{k}\Delta t}-1)$$

where $v_{\mathrm{jk}}$ is the rate of uptake or secretion specified by the FBA solution for that species and metabolite at that time point, μ*_k_* is the species growth rate, bio*_k_*(*t_n_*) is the species abundance, and Δ*t* is the size of the time step. Finally, combining the flux solutions of all species, nutrient inflow, and dilution, along with the steady-state assumption of no intracellular metabolite accumulation, the concentration of a given metabolite in the shared nutrient environment at the next time point, *M_j_*(*t_n+1_*) can be updated as follows:

$$M_{j}\left(t_{n+1}\right)=M_{j}\left(t_{n}\right)+m_{\text{FBA}}^{j}\left(t_{n}\right)+m_{\text{in}}^{j}\Delta t-M_{j}\left(t_{n}\right)D\Delta t$$

where $m_{\text{FBA}}^{j}\left(t_{n}\right)$ is the metabolic impact from all species (considering their abundance and the uptake and secretion rates of metabolite *j*) and $m_{\text{in}}^{j}$ is the inflow rate of metabolite *j*. This process of calculating uptake rates, flux balance analysis solutions, and updated metabolite concentrations was then repeated iteratively for the duration of the simulation.

Each simulation was run for a period of 144 h or 576 time steps (with the exception of the analyses of various simulation durations presented in Fig. S7A to E in the supplemental material). This time period was long enough for most simulation runs to begin to approach a steady-state composition without fully converging. Specifically, in >65% of the simulations analyzed in our study, the change in abundance in any species over the final 3 h was less than 0.01% of the carrying capacity (see below), and none had changes greater than 0.3% of the capacity over that period. The concentrations of species and metabolites, the species growth rates, and the solved rates of all reactions for each species (including uptake and secretion) were recorded for each step of each simulation and used for subsequent analyses.

### Simulation initialization parameters.

For the 10-species data sets, we fixed the initial total abundances of microbes to the carrying capacity for the given system and medium, which was estimated to be 0.433 units of biomass. This capacity was calculated as the average final total abundance from a set of simulations with various compositions and low initial abundances. We then adjusted the relative abundances, increasing the abundance of one species at a time at the expense of all other species equally. Specifically, for each species, we ran simulations in which the ratios of that species’ initial abundance to all other species were 2, 3, 4.5, 6, 9, and 13 (equating to a range in relative abundance of 10% to 60% for each species). This resulted in a total of 61 simulation runs (one with all species starting at equal abundances and 6 with increased abundance of each species). We chose this sample size to approximately represent the sample sizes of published cross-sectional microbiome-metabolome association studies (17, 18). We set the initial inflow concentrations to the amount of dilution that would occur over 1 h under the calculated inflow rates.

### Calculation of contribution values for variable metabolites.

We calculated contribution values for all metabolites with a variance in concentration above the 25th percentile. We chose this threshold in order to include as many metabolites as possible while excluding those that showed variation in only half or fewer of the simulation runs or whose variation would be subject to numerical errors.

### Comparison with Shapley values.

We implemented an approximate Shapley value algorithm (50) as an alternative strategy to calculate the contributions for the main 10-species simulated data set. Briefly, 15,000 random orderings of the 10 species were randomly generated. For each ordering, the variance in metabolite activity was calculated for subsets of size 1 to size 10, adding species according to the specified ordering. The difference in variance as a given species was added to the subset, denoting the marginal contribution of that species to variation, was recorded. The average marginal contribution across all orderings for each species was then defined as its contribution to variance.

### Species-metabolite correlation analysis.

We calculated Spearman correlations between absolute species abundances (quantified as total biomass) and concentrations of variable metabolites. We used absolute abundances in order to evaluate the relationships between species and metabolites under the hypothetically best possible measurements of both data types. We also compared correlation results using relative abundances and found very minimal differences in the main 10-species simulation data set; only 7 species-metabolite pairs (1.3%) were found to be significantly correlated using absolute abundances but not relative abundances, and only 4 pairs (0.8%) were found to be correlated using relative abundances but not absolute abundances.

We used a *P* value threshold of 0.01 to classify “significant” associations for binary comparisons. For interpretability, we refer to *P* values not corrected for multiple-hypothesis testing, since the number of tests remained constant across most analyses (520 possible species-metabolite pairs). The 0.01 threshold that we use to define significantly correlated pairs is equivalent to a Benjamini-Hochberg corrected false-discovery threshold of 0.027, calculated using the R function *p.adjust* (86). We used this false-discovery threshold as the cutoff for the analysis of correlations within subgroups.

### Logistic regression modeling of correlation outcomes.

We used logistic regression models to identify factors that can be used to predict whether a non-contributing species-metabolite pair displayed a significant correlation (false positive) and whether a key contributor species-metabolite pair failed to be correlated (false negative). We used the *glm* function in R to fit models of the log odds of whether a non-contributing species was correlated with its corresponding metabolite (false positive or true negative), using as predictors grouped indicator values for species and metabolite identities. We separately fit another set of logistic regression models to predict whether a key contributor species is correlated (true positive or false negative) using the same predictors. Models were compared using likelihood ratio tests and the *anova* function in R.

### Simulations with various inflow quantities.

We ran 8 additional sets of simulations with the same set of 61 different initial species compositions but with various degrees of inflow fluctuations. Specifically, the nutrient inflow quantities were sampled independently from a normal distribution, with a mean of the original inflow concentration and a standard deviation equal to a set percentage of the mean. The 8 levels of deviation were 0.5%, 1%, 2%, 3%, 4%, 5%, 8%, and 10%. In the comparison of correlation results across samples, we evaluated the same set of 52 variable metabolites as were used for the original data set for consistency, although, given the added stochasticity, additional metabolites met the same variance cutoff as we used to define variable metabolites.

To evaluate correlation performance as a function of increasing environmental contribution, we binned the 38 analyzed inflow metabolites across the 8 data sets on the basis of the size of the environmental contribution to variance for the metabolite in that data set. In other words, metabolites in any data set with an environmental contribution of greater than 0 but less than 10% of the total positive variance contributions were binned into a single category, those with an environmental contribution of between 10% and 20% were binned into the next category, and so on. We analyzed the 52 metabolites in the original constant-environment data set as a separate category and did the same for the 14 metabolites not present in the inflow in each of the 8 environmentally differing data sets.

Confidence intervals for AUC values were calculated using the *pROC* package in R (87) and a bootstrap method with 500 resamplings.

### Simulations of Human Microbiome Project-based microbiota.

To simulate the more complex gut microbiota, we downloaded the 16S rRNA sequence variant abundance tables from the Human Microbiome Project (61), processed with deblur (88), from Qiita (89). We also downloaded rRNA sequences for all of the 818 genomes corresponding to AGORA v1.0.2 models from NCBI RefSeq and GenBank using the biomartr R package (90). We used *vsearch* version 2.8.1 (91) to map the HMP sequences to the AGORA ribosomal sequences with 97% identity, with the *max_rejects* parameter set to 0 in order to obtain the highest identity match for each sequence variant. We chose to model a subset of 57 samples for which at least 25% of their total read counts successfully mapped to an AGORA genome. We normalized species abundances based on the 16S rRNA copy number of the corresponding genome and initialized 57 simulations with the starting relative abundances determined based on the AGORA-mapped relative abundances of these samples. We updated the nutrient inflow to enable growth by most models as follows. We assessed whether the addition of each individual metabolite to the original nutrient inflow had a growth-promoting effect on any of the species, specifying quantities similar to those of the average European diet in the Virtual Metabolic Human database where possible (92). Metabolites that promoted growth in at least one species were retained in the revised nutrient inflow, and the process of testing for increased growth with the addition of any single metabolite was repeated. After two rounds of addition of metabolites to the inflow, 15 models, representing 3.4% of the total normalized abundance across all samples, still displayed zero growth. We removed these from the simulations and used the final updated nutrient inflow with the 131 remaining models. All other simulation parameters were the same as for the 10-species community simulations. In our analyses of the role of interspecies correlation in this data set, we excluded species that appeared in fewer than 4 samples.

### Application of MIMOSA to simulated data and comparison with correlation analysis.

We applied MIMOSA v1.0.2 (github.com/borenstein-lab/MIMOSA) (65) to the obtained set of metabolite and species abundances for the main 10-species data set. To construct the community metabolic network model required by MIMOSA, we merged the 10 species-level models used in the simulations into a single stoichiometric matrix. If a reversible reaction ever proceeded in only a single direction in any simulation, we encoded it as nonreversible. To apply the KEGG-based version of MIMOSA, we converted the model metabolite identifiers (IDs) to KEGG IDs (63), downloaded KEGG Orthology gene annotations for the 10 modeled species from the IMG/M database (84), and ran a MIMOSA analysis using the KEGG metabolic network model encoded in *reaction_mapformula.lst* (KEGG version downloaded February 2018).

### Data availability.

Code for all the analyses presented in this study is available online in the form of R notebooks at https://github.com/borenstein-lab/microbiome-metabolome-evaluation. The code and media files for performing dynamic FBA coculture simulations are available from http://borensteinlab.com/download.html. Simulation data generated and analyzed in this study and displayed in the figures are also available for download from http://borensteinlab.com/download.html.
