# Supplementary material for: Defining and Evaluating Microbial Contributions to Metabolite Variation in Microbiome-Metabolome Association Studies
Source: mSystems. 2019 Dec 17;4(6):e00579-19. doi: 10.1128/mSystems.00579-19 (PMC6918031; doi:10.1128/mSystems.00579-19)
Supplement: TEXT S1 [file mSystems.00579-19-s0001.pdf]

Supplementary Results

Simulated species responses to media variation recapitulate experimental results

We ran several sets of simulations with the same set of 61 initial species compositions as the main 10-species dataset but with small amounts of stochastic noise added to the nutrient inflow. Specifically, inflow concentrations for each compound in each simulation were sampled from a normal distribution with a mean equal to the compound's original inflow rate and a standard deviation set to a particular fraction of the mean (Methods). We then examined whether the differences in simulated species growth across simulations with large media fluctuations (8-10%) recapitulated experimental observations from Faith *et al.* (1). Overall, we found agreement in several observed trends. First, Faith *et al.* observed that casein abundance was strongly associated with the total community biomass (presumably, due to limiting amino acid or nitrogen concentrations). In our simulations, the total amount of amino acids is indeed positively associated with total biomass (Spearman rho=0.25, *p*=0.005). Moreover, we fit linear regression models of total biomass across all simulations based on inflow metabolite levels, and found that concentrations of both amino acids and carbohydrates explain most of the variation in this quantity in our simulations (adjusted R^2 of 0.97), with other nutrients adding only negligible effects, similar to the results of Faith *et al*. Faith *et al.* further observed that while most species increase their growth rate in the presence of higher protein; *E. rectale*, *D. piger* and *M. formatexigens* are negatively associated with the amount of casein in the mouse diet. In our simulations, a model predicting growth rate based on amino acid levels explains no variation in *E. rectale* and only 8% in *M.*

formatexigens, in contrast to other taxa, for which amino acids explained up to 54% of growth rate variation. Additionally, the initial growth rates for all species were positively correlated (Spearman, $p < 0.1$) with an average of 2.7 different amino acid compounds (range 1 to 6), while *E. rectale* and *M. formatexigens* were each only correlated with a single amino acid, *L*-cysteinylglycine. Lastly, our simulations also recapitulate differences in carbohydrate use: Faith *et al*. observed preferential expansion of *B. ovatus* and *B. thetaiotaomicron* on a high-starch diet compared to a high-sugar diet. In our simulations, the growth rate of all species was associated with the amount of available simple sugars, but only *B. ovatus*, *B. thetaiotaomicron*, and *E. rectale* were significantly correlated with the quantity of starch in the inflow (Spearman rho coefficients of 0.5, 0.53, 0.53 respectively, all $p < 10^{-9}$). These findings suggest that our simulation framework successfully encapsulates at least some of the nutrient limitations shaping the growth dynamics of this model community.

Analysis of an alternative definition of contribution values based on steady-state fluxes

Our contribution value metric attributes metabolite variance to each species depending on its cumulative metabolite uptake or secretion over the entire simulation, rather than its arrived-at steady-state metabolite flux at the time of “sampling”. To assess the impacts of this choice, we calculated contribution values using an alternative definition based solely on steady-state fluxes. Specifically, we calculated the contribution of each species to the metabolite flux at the final time point of simulations run for 144 hours and for 1440 hours, using the same 61 initial species compositions as the main 10-species dataset and identical nutrient inflow. Under this definition, steady-state contribution

values explain the variation in metabolite flux rate at the time of sampling, rather than the accumulated variation in metabolite concentrations (cumulative contribution values). We compared these alternative steady-state contributions to the original set of cumulative contribution values at both time points, finding that they are highly similar. Specifically, in our original dataset of simulations run for 144 hours, the Pearson correlation between steady-state and cumulative contribution values for each metabolite was on average 0.99 (minimum of 0.75, median of 0.999 across all metabolites). Only 6 of the 520 analyzed species-metabolite pairs differ in contributor status between the two definitions: 4 pairs are key cumulative contributors but not steady-state contributors, and 2 pairs are the reverse. The AUC for detection of steady-state contributors is 0.710 (compared with 0.717 for cumulative contributors). These differences were even more negligible for simulations run for longer durations: in a dataset of simulations run for 1440 hours, the average metabolite-level correlation between steady-state and cumulative contribution values was 0.9999 (minimum 0.9997, median of 0.99999). These results indicate that for these simulations, differences in species composition and metabolic activity early in the simulation are *not* the predominant factor in the observed discrepancy between species-metabolite correlations and true key contributors to metabolic variation.

Analysis of an alternative definition of key taxon-metabolite pairs

For most analyses, we defined the key taxonomic contributors for a particular metabolite as those species with the highest positive contribution values, or those that are responsible for the observed pattern of variation in a metabolite. However, an alternative goal could be to detect all microbes that substantially impact levels of a given

metabolite across samples, regardless of whether their effects are ultimately reflected in the observed concentrations. To this end, we defined *key player* species as those with a contribution value, either positive or negative, greater in magnitude than 20% of the total contribution magnitude. This resulted in 91 species-metabolite key player pairs, including 65 of the previously defined ‘positive’ key contributor pairs but also 26 players with negative contributions, which were distributed similarly across metabolites and species (Figure S5, panels A-B). Examining how well these key players were detected by a correlation-based analysis, we found similar performance as for key contributors (Figure S5, panels C-G), including a comparable positive predictive value (31.9%) and AUC (0.73).

Effects of simulation length and* Vmax *parameter on correlation results

We assessed the sensitivity of our correlation results to the parameters used in our simulations. Specifically, we first evaluated the effect of the duration of simulations on our results. We ran additional simulations of the same 61 10-species compositions for 5,760 time points (or 1,440 hours), and calculated contribution values and correlation coefficients at 22 intermediate time points starting at 36 hours (Figure S7). Species compositions and metabolite concentrations became increasingly less variable with longer simulation time, converging towards similar steady states dominated in abundance by 5 of the 10 species (Figure S7A-B). Correspondingly, the number of key contributors decreased with increasing simulation length, from 121 contributors across all 52 analyzed metabolites at 36 hours, to 75 at 1,440 hours (Figure S7B-C). The number of significantly correlated species-metabolite pairs, however, increased from 179 to 375 over the same datasets, detecting contributors with higher sensitivity but

lower specificity (Figure S7D). Ultimately, the AUC and positive predictive value both decrease slightly with increasing simulation length, with the AUC shifting from 0.67 to 0.73 and positive predictive value from 39.7% to 18.4% (Figure S7E). This transition occurs sharply initially before reaching an inflection point and beginning to stabilize around 144 hours, the length of time chosen for our main analysis.

We also generated additional datasets with the same initial species compositions but with widely varied values for the universal *Vmax* parameter, which was set to 20 in the main set of analyses. Changing this parameter had very minimal impact on both the simulation abundance profiles and the results of correlation analysis (Figure S7F-H). The AUC for the identification of key contributors was not associated with the value of the Vmax parameter, and only ranged from 0.70 to 0.72.

Features distinguishing true key contributors from false positives among correlated pairs

We constructed additional regression models to assess whether there are features that can distinguish true key contributors from false positives among all correlated species-metabolite pairs. We fit regression models to similarly assess whether species and/or metabolite identity are indicative of whether a correlated species-metabolite pair represents a true or false positive relationship. We found that species identity (p = 0.047), but not metabolite identity, was predictive of key contributor status among correlated pairs. This is unsurprising given that the number of key contributions from each species varied widely, while all metabolites have at least one key contributor.

Additional effects of inflow fluctuations on contribution and correlation profiles

We assessed whether the addition of external metabolite fluctuations impacted the profile of species contributing to each metabolite. For most metabolites (28 out of 52, including 12 out of 14 non-inflow metabolites), the top microbial contributor did not change across all levels of fluctuation. However, for many inflow metabolites, the large external fluctuations can result in a switch of contribution value sign. In these cases, activity by a microbe that contributed to variation in a constant-inflow setting instead has a mitigating impact, resulting in a negative contribution. Of the 65 key contributors to variation in inflow metabolites in the original dataset, 34 (52%) had a negative contribution value in at least one simulation run with external fluctuations. This observation highlights that our definition of key contributors is context-dependent, identifying the entities primarily responsible for the observed variation.

We also examined whether the detection of the 14 variable metabolites not present in the nutrient inflow was affected by random fluctuations in inflow metabolites. Variation in 8 of these metabolites was significantly positively correlated with variation in the surrounding inflow (Spearman rho, $p < 0.01$), suggesting that their synthesis fluxes were affected by changes in microbial growth or nutrient usage that resulted from environmental shifts. Correlation analysis tended to identify key contributors for these metabolites with slightly higher specificity and lower sensitivity as inflow fluctuations increased (Figure S8).

Supplementary References

1. Faith JJ, McNulty NP, Rey FE, Gordon JI. 2011. Predicting a Human Gut Microbiota's Response to Diet in Gnotobiotic Mice. Science 333:101–104.
